# Validating the Calgary Simulation Curriculum: A Retrospective Review of Face and Content Validity of a Surgical Simulation Curriculum in Otolaryngology—Head and Neck Surgery

**DOI:** 10.1177/19160216261443996

**Published:** 2026-04-27

**Authors:** Daniel Ghods-Esfahani, Fatemeh Ramazani, Justin Lui

**Affiliations:** 1 Division of Otolaryngology—Head and Neck Surgery, Department of Surgery, University of Alberta, Edmonton, AB, Canada; 2 Ohlson Research Initiative, Arnie Charbonneau Cancer Institute, Cumming School of Medicine, University of Calgary, Calgary, AB, Canada; 3 Section of Otolaryngology—Head and Neck Surgery, Department of Surgery, University of Calgary, Calgary, AB, Canada

**Keywords:** simulation curriculum in otolaryngology, residency training curriculum, face validity of a simulation curriculum, content validity of a simulation curriculum

## Abstract

**Importance:**

Simulation is a useful educational adjunct in surgical training. While a variety of simulation models exist in Otolaryngology—Head and Neck Surgery (OHNS), no multi-session curriculum, encompassing a breadth of educational objectives, has been described or validated.

**Objective:**

The objective of this study was to determine the face and content validity of an OHNS simulation curriculum, including preparatory materials, session content, and method of content delivery (cadaveric and high-fidelity mannequin simulation).

**Study Design:**

Mixed-methods validation study.

**Setting:**

University of Calgary (UofC) OHNS Residency Program.

**Participants:**

UofC OHNS residents enrolled in the program from 2022 to 2024.

**Interventions:**

Retrospective review of session content and feedback following completion of the Calgary Simulation Curriculum (CSC) by UofC OHNS resident, in addition to curriculum mapping of session objectives to OHNS training objectives, established by the Royal College of Physicians and Surgeons of Canada (RCPSC).

**Main Outcome Measures:**

Face validity was assessed through resident feedback scores on the overall educational value, teaching quality, and quality of preparatory materials of the CSC sessions. Content validity was assessed through the curriculum mapping process of session objectives to the RCPSC training objectives.

**Results:**

CSC is comprised of 5 simulation sessions which include: management of pediatric airway foreign bodies, neck dissection, nasal fractures, sphenopalatine artery ligation, endoscopic sinus surgery, and rhinoplasty. Curricular mapping demonstrated strong face validity of the curriculum as all sessions aligned well with the RCPSC outline competencies. The curriculum also demonstrated overall strong content validity as evidenced by high perceived educational value, quality of teaching, as well as preparatory materials.

**Conclusions:**

The CSC demonstrated both face and content validity and represents a valuable structured simulation-based educational tool for Canadian OHNS programs. This curriculum can be used as a complementary adjunct to surgical postgraduate training as a formative or summative educational tool for trainees.

## Graphical abstract

**Graphical Abstract:**
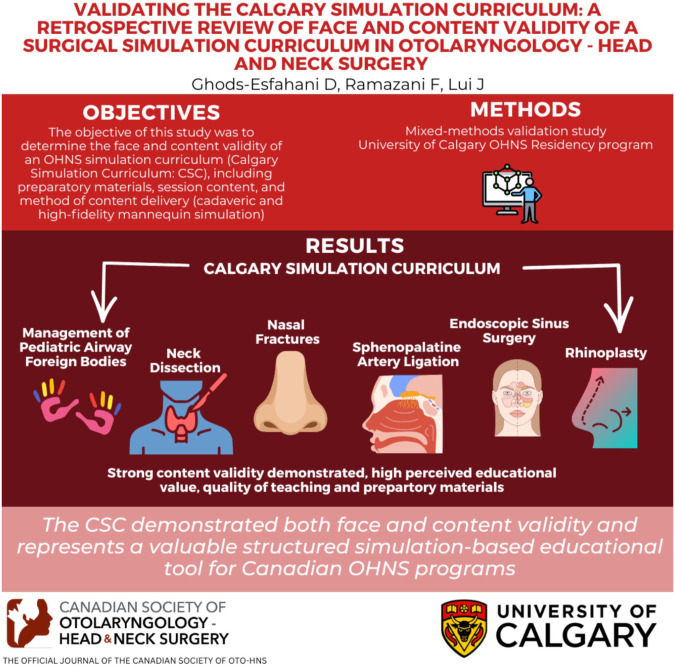

## Key Messages

- The Calgary Simulation Curriculum (CSC) is a validated, multi-session simulation curriculum integrating cadaveric and mannequin-based models across core Otolaryngology—Head and Neck Surgery competencies.
- Iterative evaluation and alignment with the Royal College of Physicians and Surgeons of Canada Entrustable Professional Activities support the CSC’s feasibility, educational value, and transferability within competency-based residency training.

## Introduction

Surgical residency training has long been centered around role modeling and experiential learning, with technical skills taught under the direct supervision of attending surgeons. Increased case times with attributable increases in costs, time constraints, and complications related to operative time highlight inefficiencies of a traditional surgical education model.^1,2^ Current theories of experiential and adult learning emphasize the importance of sustained, deliberate exposure to a given procedure within a safe environment that simultaneously values learner independence.^3^ As a result, simulation has been used as an adjunct to direct clinical interactions across a variety of different surgical residency programs to provide learners with a structured and low-stakes training environment without the risk of adversely impacting patient outcomes. Additionally, simulation curricula offer learners a structured scope and exposure to procedural competencies in a deliberate manner, rather than random case exposure in traditional surgical education paradigms.

Data thus far on the utility of simulation in surgical education has been positive. A study by Kantar et al demonstrated that plastic surgery residents who underwent a digital and haptic surgical simulator had improved acquisition of surgical skills in comparison to residents who relied on textbooks for this knowledge.^4^ Furthermore, Dean et al identified increased surgical competency and self-reported confidence among ophthalmology residents who underwent a 5 day simulation-based training course.^5^ Within this study, the group of trainees in the experimental simulation group went on to complete a greater number of surgeries 1 year post-completion of the course, which suggests that incorporation of simulation in surgical training provides a sustained impact on learner confidence and competence.^5^

Current literature also reports positive resident perspectives toward the use of simulation. Chow et al report that surgical residents appreciate surgical simulation as a useful adjunct to clinical training.^6^ A study by Bodani et al demonstrated increased self-reported confidence in residents’ approach to endoscopic neurosurgery as well as a significant interest in future simulation sessions among neurosurgery residents.^7^ Similarly, a study of Otolaryngology—Head and Neck Surgery (OHNS) residents demonstrated an increase in residents’ self-perceived level of experience and comfort with facial fractures following a 1 day cadaveric simulation course on facial trauma.^8^ Comparably, Obstetrics and Gynecology residents who underwent a simulation course prior to partaking in a live laparoscopic salpingectomy procedure demonstrated improvement in procedural skills, as well as self-reported comfort with the procedure.^9^

A systematic review into the current available surgical simulation models in OHNS by Musbahi et al demonstrated a total of 64 available surgical simulation models, 59 of which of had at least 1 validation study.^10^ All the simulation models described in this study pertained to a single operative procedure. To date, no formal curriculum encompassing more than 1 subspecialty within OHNS has been described or validated. Given the scope and breadth of OHNS as a surgical specialty, with a wide array of procedures and surgical tools, there is a clear need for a comprehensive surgical simulation curriculum to help residents achieve operative competence across the various subspecialties of OHNS.^1^

The primary objective of this study was to evaluate the face validity and perceived educational utility of a dedicated OHNS simulation curriculum, the Calgary Simulation Curriculum (CSC), as assessed by resident feedback. The secondary objective was to assess the content validity of the CSC through alignment with competencies outlined by the Royal College of Physicians and Surgeons of Canada (RCPSC) for OHNS.

## Materials and Methods

### Curriculum Development and Needs Assessment

The CSC was developed using a learner-centered, iterative process aimed at addressing perceived gaps in OHNS residency training that were not consistently encountered in clinical practice. Curriculum topics were identified through structured discussions involving senior resident representatives and faculty members who serve on the residency program committee. These discussions focused on areas where residents reported limited operative exposure, high clinical complexity, or low tolerance for error in real-world settings.

Proposed simulation sessions were reviewed collaboratively with resident representatives to assess perceived educational value and feasibility. Consensus regarding the utility of each proposed session was achieved among a group of residents prior to curriculum planning and execution. This process informed the selection of 5 simulation sessions spanning multiple OHNS subspecialties.

Each session was subsequently mapped to the RCPSC Entrustable Professional Activities (EPAs) to establish content validity and ensure alignment with competency-based medical education requirements. EPAs were identified and outlined by the 2022 version of the “Entrustable Professional Activities (EPA) Guide: Otolaryngology—Head and Neck Surgery” published by the RCPSC.^11^

### Curriculum Structure and Delivery

The CSC consists of 5 simulation sessions delivered annually during protected academic time. Each annual delivery of the full 5-session curriculum was defined as 1 “iteration.” All residents participating in a given iteration completed all 5 sessions. Residents could therefore participate in multiple iterations as they progressed through training, with new postgraduate year 1 residents entering the curriculum annually. The sessions offered to residents as a part of this curriculum are listed below:

- Pediatric airway foreign body (PAFB)
- Neck dissection (ND)—levels I to IV
- Complete functional endoscopic sinus surgery (FESS)
- Nasal fracture and sphenopalatine artery ligation (NF/SPAL)
- Rhinoplasty

Simulation modalities included cadaveric dissection and mannequin-based models, selected based on the procedural requirements of each session. Sessions were facilitated using a mixed teaching model involving faculty surgeons and senior residents. Faculty members provided procedural oversight and contextual teaching, while senior residents supported near-peer instruction and intra-session guidance.

Each session was supported by a standardized dissection manual outlining learning objectives, anatomical scope, required equipment, and stepwise procedural guidance. These manuals were distributed to residents in advance to support preparation. Representative dissection manuals are provided as a Supplemental Appendix to illustrate session structure and support reproducibility.

### Evaluation and Data Collection

Evaluation of the CSC focused on establishing face validity through learner-reported outcomes. Feedback data were collected retrospectively from an internal residency program feedback platform across annual iterations of the curriculum (2022-2024). Participants included current trainees within the University of Calgary (UofC) OHNS residency training program, ranging from postgraduate years 1 to 5. No formal objective technical skill assessments (eg, Objective Structured Assessment of Technical Skills or procedural checklists) were employed as part of this initial evaluation.

Following each simulation session, residents completed structured feedback surveys using a 5-point Likert scale. Survey domains included perceived educational value, adequacy of preparation, quality of instruction, and realism of the simulation experience. A 5-point Likert Scale was used to evaluate residents’ attitudes toward the CSC across the evaluation domains. Responses were scored from 1 to 5, with scores of 5 indicating strong satisfaction and 1 indicating strong dissatisfaction. Survey responses were analyzed descriptively. Likert scale responses are reported as medians with median absolute deviation values reported in brackets. Additional analysis was performed to determine the added educational value of learning objectives and preparatory material by comparing the 3 iterations of the curriculum. A comparison was also carried out between the PAFB session and the remainder of the sessions to assess residents’ assessment of the educational value and utility of a mannequin-based simulation session to cadaveric ones.

## Results

Results are presented in 2 phases. Phase I describes the structure of the CSC and its alignment with RCPSC EPAs, including session content and mapped learning objectives (Tables 1 and 2). Phase II summarizes resident participation and feedback across 3 iterative administrations of the curriculum, including assessments of educational value, teaching quality, preparatory materials, and learning objectives (Tables 3–5).

**Table 1.** Curriculum Map of the CSC, Demonstrating Each Session’s Corresponding EPAs as Well as Each Corresponding Learning Objective Covered by the Sessions.

PAFB	ND	FESS	Rhinoplasty	NF/SPAL
Foundations EPA 9	Core EPA 13	Core EPA 9 (parts B and C)	Core EPA 18 (parts A and B)	Foundations EPA 3 (parts A and B)
ME 1.7 ME 2.1 ME 2.2 ME 2.4 ME 3.4	ME 1.4 ME 2.2 ME 3.1 ME 3.4	ME 1.4 ME 2.2 ME 3.1 ME 3.4	ME 1.4 ME 2.2 ME 3.1 ME 3.4	ME 1.4 ME 1.5 ME 2.2 ME 2.4 COM 2.1 COM 2.2 ME 3.1 ME 3.4
Core EPA 7				Foundations EPA 7 (parts A and B)
ME 1.4 ME 3.1 ME 3.4				ME 1.4 ME 1.5 ME 2.2 ME 3.1 ME 3.4
				Core EPA 8 (parts A and B)
				ME 1.4 ME 1.5 ME 2.1 ME 3.1 ME 3.4

Abbreviations: COL, collaborator; COM, communicator; CSC, Calgary Simulation Curriculum; EPA, Entrustable Professional Activity; FESS, functional endoscopic sinus surgery; ME, medical expert; ND, neck dissection; NF/SPAL, nasal fracture and sphenopalatine artery ligation; PAFB, pediatric airway foreign body.

**Table 2.** CSC Session Outline and Design. Table Reflects Initial Implementation; Subsequent Iterations Modified Preparatory Materials and Objectives as Described in “Results” Section.

Simulation characteristics	PAFB	ND (I-IV)	FESS	Rhinoplasty	NF/SPAL
Number of sessions	1	2	1	1	1
Setting	Operating room	ATSSL	ATSSL	ATSSL	ATSSL
Learning objectives	Not provided	Provided	Not provided	Not provided	Provided
Preparatory materials	Not provided	Provided	Not provided	Provided	Provided
Models	Mannequins	Cadavers	Cadavers	Cadavers	Cadavers
Supervision	Two OHNS staff, 1 anesthetist, representatives from KidSIM	Two fellowship trained OHNS oncologic surgeons	Two OHNS staff, one of which was a fellowship trained rhinologist	One fellowship trained OHNS facial plastics surgeon	One fellowship trained OHNS rhinologist
Structure	Four juniors paired with 4 senior residents	Four juniors paired with 4 senior residents	Four juniors paired with 4 senior residents	Four juniors paired with 4 senior residents	Four juniors paired with 4 senior residents
Session design	Imaging review and case discussion Simulated removal by junior residents, guided by seniors. Mannequin response controlled by KidSIM	Junior resident guided dissection by senior residents with concurrent staff supervision	Senior resident demonstrating dissection followed by junior residents carrying out dissection on the contralateral side	Stepwise demonstration by attending physician with pauses for resident pairs to carry out dissection	Junior resident guided dissection by senior residents with concurrent staff supervision

Abbreviations: ATSSL, Advanced Technical Skills Simulation Lab at the University of Calgary; CSC, Calgary Simulation Curriculum; FESS, functional endoscopic sinus surgery; KidSIM, Pediatric Simulation Program at Alberta Children’s Hospital; ND, neck dissection; NF/SPAL, nasal fracture and sphenopalatine artery ligation; OHNS, Otolaryngology—Head and Neck Surgery; PAFB, pediatric airway foreign body.

**Table 3.** Resident Feedback Data—Median (Median Absolute Deviation) of Resident Feedback Data for the First and Third Iteration of the CSC.

Evaluation metrics	PAFB		ND		Rhinoplasty		NF/SPAL		FESS	
	Iteration 1 (N = 10)	Iteration 3 (N = 7)	Iteration 1 (N = 10)	Iteration 3 (N = 8)	Iteration 1 (N = 9)	Iteration 3 (N = 8)	Iteration 1 (N = 10)	Iteration 3 (N = 8)	Iteration 1 (N = 10)	Iteration 3 (N = 8)
Educational value	4.0 (0.5)	4.0 (1.0)	5.0	4.5 (0.5)	5.0	4.5 (0.5)	4.5 (0.5)	4.5 (0.5)	4.0 (1.0)	5.0
Quality of faculty teachers	5.0	4.0	5.0	4.5 (0.5)	5.0	4.5 (0.5)	5.0	5.0	5.0	5.0
Quality of resident teachers	5.0	5.0	5.0	5.0	5.0	5.0	5.0	5.0 (0.5)	5.0	5.0
Quality of teaching material	3.5 (0.5)	5.0	4.5 (0.5)	5.0	4.0	5.0	4.0 (1.0)	5.0	4.0	5.0

Abbreviations: FESS, functional endoscopic sinus surgery; ND, neck dissection; NF/SPAL, nasal fracture and sphenopalatine artery ligation; PAFB, pediatric airway foreign body.

**Table 4.** Residents’ Assessment of Session Learning Objectives During the First Iteration of the CSC. Percentages Calculated Based on Respondents Who Attended Each Session.

Session	Learning Objectives			
	Met	Not met	Not provided	Did not attend
PAFB	4 (44%)	1 (11%)	4 (44%)	1
ND	7 (78%)	0	2 (22%)	1
Rhinoplasty	2 (25%)	0	6 (75%)	1
NF/SPAL	5 (63%)	0	3 (37%)	2
FESS	4 (40%)	1 (10%)	5 (50%)	0

Abbreviations: CSC, Calgary Simulation Curriculum; FESS, functional endoscopic sinus surgery; ND, neck dissection; NF/SPAL, nasal fracture and sphenopalatine artery ligation; PAFB, pediatric airway foreign body.

**Table 5.** Median Scores for the PAFB Session Before and After the Addition of Preparatory Materials.

Evaluation metrics	Iteration 1 PAFB without preparatory material (MAD)	Iteration 2 PAFB with preparatory material (MAD)
Number of respondents	10	7
Educational value	4.0 (0.5)	5.0
Quality of teaching: faculty	5.0	5.0
Quality of teaching: residents	5.0	4.0
Quality of teaching: material	3.5 (0.5)	5.0

Abbreviations: MAD, median absolute deviation; PAFB, pediatric airway foreign body.

### Phase I: Content Validity

The CSC consists of 5 simulation sessions spanning multiple subspecialties within OHNS. Mapping of curricular content to RCPSC EPAs demonstrated alignment across multiple stages of competency-based training (Table 1).

The PAFB and NF/SPAL sessions aligned with both Foundations of Discipline and Core of Discipline EPAs, whereas the ND, FESS, and rhinoplasty sessions primarily aligned with Core of Discipline EPAs. Across all sessions, mapped learning objectives predominantly addressed disease pathophysiology as well as medical and surgical management. Objectives related to patient communication and interdisciplinary collaboration were not explicitly addressed within the CSC. Details of session structure, instructional modality, simulation models, and preparatory materials are summarized in Table 2.

### Phase II: Resident Participation

A total of 25 residents provided feedback across 3 annual iterations of the CSC. Ten out of 10 eligible residents participated in the first iteration, 7 out of 8 eligible residents participated in the second, and 8 out of 8 eligible residents participated in the third. All residents who participated in the sessions completed the survey. In the second iteration, 6 residents had previously completed the first iteration, while 1 incoming resident participated in the curriculum for the first time. In the third iteration, 5 residents had completed both prior iterations, 1 resident had completed only the second iteration, and 2 incoming residents participated in the curriculum for the first time. During the first iteration of the curriculum, sessions delivered without preparatory materials demonstrated comparatively lower ratings for teaching materials, most notably the PAFB and FESS sessions (Table 3). In contrast, sessions supported by structured learning objectives and preparatory materials in subsequent iterations demonstrated uniformly high ratings across all evaluated domains.

In the second iteration, data were collected for the PAFB and ND sessions only, both of which included preparatory materials and learning objectives. Median ratings for educational value, quality of teaching materials, and faculty teaching were high for both sessions, while ratings for senior resident teaching remained favorable (Table 5). During the third iteration, all 5 sessions included preparatory materials and learning objectives, and ratings across all domains were consistently high.

### Assessment of Learning Objectives

Resident assessment of session learning objectives during the first iteration of the curriculum is summarized in Table 4. Despite the presence of predefined learning objectives for some sessions, a proportion of residents reported being unaware of these objectives. This was observed most prominently in the NF/SPAL and ND sessions. For sessions in which learning objectives were not provided, residents reported variable perceptions of whether session goals were met.

### Qualitative Feedback

Qualitative feedback from residents reflected an overall positive view of the CSC. During the first iteration, residents frequently requested additional preparatory materials, particularly for the PAFB session. Concerns regarding simulation realism were also noted, including limitations related to mannequin fidelity and variability in cadaver quality. In subsequent iterations, residents suggested the inclusion of additional pediatric cases, increased supervision, and the incorporation of visual aids within dissection manuals.

## Discussion

Over the past decade, surgical simulation has emerged as an important educational adjunct aimed at addressing limitations of traditional apprenticeship-based surgical training while enhancing resident confidence and preparedness for operative practice.^1,5,8,12^ Within OHNS, simulation initiatives have largely focused on isolated procedures rather than comprehensive curricula spanning multiple subspecialties. In response to this gap, the CSC was developed at the UofC in 2022 as a structured, multi-session simulation program incorporating head and neck oncology, rhinology, pediatrics, and facial plastic surgery.

To meaningfully contribute to residency education, a simulation curriculum must demonstrate educational validity.^13^ To our knowledge, this study represents the first published evaluation of both content and face validity of a multi-subspecialty simulation curriculum in OHNS. By explicitly aligning simulation content with RCPSC EPAs and evaluating learner perceptions across multiple iterations, the CSC provides a reproducible framework for simulation-based training within a Canadian competency-based medical education context.

Content validity was supported through systematic mapping of CSC sessions to EPAs across the Foundations of Discipline and Core of Discipline stages of training. This mapping demonstrates relevance for both junior and senior residents and supports the CSC’s role as an educational adjunct addressing competencies that may be inconsistently encountered in clinical practice. Intentional variation in the number and breadth of mapped objectives across sessions reflects deliberate curriculum design rather than omission. Sessions such as NF/SPAL were designed to address both foundational and advanced competencies, whereas sessions such as FESS and ND targeted more focused senior-level technical skills.

Face validity was assessed through resident feedback across 3 annual iterations of the curriculum. Overall, residents consistently rated the CSC highly with respect to educational value, quality of instruction, and realism. High ratings for both faculty and senior resident teaching across all iterations suggest that a mixed faculty-resident teaching model can be effective and may enhance feasibility and sustainability, particularly in programs with limited faculty availability.

Importantly, this study also demonstrates the value of iterative curriculum evaluation as an implementation strategy. Structured resident feedback identified modifiable barriers to effective delivery, including inadequate visibility of learning objectives and variability in preparedness. Targeted adaptations—most notably the introduction of explicit learning objectives and preparatory materials—were associated with improved perceived preparedness and educational value in subsequent iterations. These findings highlight how modest, low-resource modifications can enhance acceptability and effectiveness while preserving core curriculum components. From an implementation perspective, this adaptive approach supports fidelity, scalability, and sustained integration of simulation into residency training. This approach aligns with contemporary implementation frameworks that emphasize stakeholder engagement, adaptability, and iterative refinement as key determinants of successful educational innovation.

These findings are consistent with educational theory and prior simulation literature. Prior knowledge has been shown to increase learner engagement and cognitive capacity for skill acquisition.^14^ Reznick emphasizes that early phases of technical skill development should occur outside the operating room, allowing learners to focus on cognitive understanding before procedural execution.^15^ This aligns with Fitts and Posner’s model of motor skill acquisition, in which mastery of the cognitive phase precedes integration and automation.^16^ The CSC’s preparatory materials likely facilitated this process, thereby enhancing the educational value of simulation sessions.

Resident feedback also identified areas for ongoing improvement. During the initial iteration, a proportion of residents reported being unaware of learning objectives despite their existence, underscoring the importance of clear communication and accessibility. Explicit learning objectives are known to enhance educational efficacy by clarifying expectations and guiding learner focus.^17^ Future iterations of the CSC will therefore prioritize improved dissemination of objectives and preparatory materials, potentially supported by protected preparatory time. Additionally, while teaching quality was consistently rated highly, the development of standardized instructor manuals may further support consistency and sustainability, a strategy supported in the educational literature.^18^

Challenges related to simulation fidelity, including mannequin realism and cadaver quality, were also identified. These limitations are well described in the literature and reinforce the role of simulation as a complement to, rather than a replacement for, clinical experience.^2^

Several limitations warrant acknowledgment. Outcomes were limited to learner-reported measures, and objective assessments of technical performance were not included. The study was conducted at a single institution, which may limit generalizability. Future work will focus on evaluating the impact of CSC participation on formal assessments, including in-training and board examination performance. Establishing such associations would provide important evidence of long-term educational impact as the curriculum continues to mature.

Collectively, these findings suggest that the CSC represents a feasible and adaptable approach to multi-subspecialty simulation in OHNS. By aligning with national competency frameworks and incorporating deliberate, iterative evaluation, the CSC shows promise as a transferable model for programs seeking to integrate structured simulation into competency-based residency training.

## Conclusion

This study describes the development and proof of concept of the CSC, a multi-session, multi-subspecialty simulation program designed to support competency-based otolaryngology residency training. Through intentional alignment with Royal College EPAs and iterative refinement informed by resident feedback, the CSC demonstrates early evidence of content and face validity. Importantly, the curriculum was designed using a learner-centered approach and implemented using a mixed faculty-resident teaching model, supporting both feasibility and sustainability. While outcomes are limited to learner-reported measures, these findings suggest that a structured simulation curriculum can enhance perceived educational value and address training gaps not consistently encountered in clinical practice. Future work will focus on integrating objective performance assessments, evaluating the impact of curriculum participation on formal examination outcomes, and exploring broader adoption across Canadian training programs. Collectively, the CSC represents a promising framework for structured simulation-based education in OHNS.

## Supplemental Material

sj-docx-1-ohn-10.1177_19160216261443996 – Supplemental material for Validating the Calgary Simulation Curriculum: A Retrospective Review of Face and Content Validity of a Surgical Simulation Curriculum in Otolaryngology—Head and Neck Surgery Supplemental material, sj-docx-1-ohn-10.1177_19160216261443996 for Validating the Calgary Simulation Curriculum: A Retrospective Review of Face and Content Validity of a Surgical Simulation Curriculum in Otolaryngology—Head and Neck Surgery by Daniel Ghods-Esfahani, Fatemeh Ramazani and Justin Lui in Journal of Otolaryngology - Head & Neck Surgery

sj-docx-2-ohn-10.1177_19160216261443996 – Supplemental material for Validating the Calgary Simulation Curriculum: A Retrospective Review of Face and Content Validity of a Surgical Simulation Curriculum in Otolaryngology—Head and Neck Surgery Supplemental material, sj-docx-2-ohn-10.1177_19160216261443996 for Validating the Calgary Simulation Curriculum: A Retrospective Review of Face and Content Validity of a Surgical Simulation Curriculum in Otolaryngology—Head and Neck Surgery by Daniel Ghods-Esfahani, Fatemeh Ramazani and Justin Lui in Journal of Otolaryngology - Head & Neck Surgery

sj-docx-3-ohn-10.1177_19160216261443996 – Supplemental material for Validating the Calgary Simulation Curriculum: A Retrospective Review of Face and Content Validity of a Surgical Simulation Curriculum in Otolaryngology—Head and Neck Surgery Supplemental material, sj-docx-3-ohn-10.1177_19160216261443996 for Validating the Calgary Simulation Curriculum: A Retrospective Review of Face and Content Validity of a Surgical Simulation Curriculum in Otolaryngology—Head and Neck Surgery by Daniel Ghods-Esfahani, Fatemeh Ramazani and Justin Lui in Journal of Otolaryngology - Head & Neck Surgery

sj-docx-4-ohn-10.1177_19160216261443996 – Supplemental material for Validating the Calgary Simulation Curriculum: A Retrospective Review of Face and Content Validity of a Surgical Simulation Curriculum in Otolaryngology—Head and Neck Surgery Supplemental material, sj-docx-4-ohn-10.1177_19160216261443996 for Validating the Calgary Simulation Curriculum: A Retrospective Review of Face and Content Validity of a Surgical Simulation Curriculum in Otolaryngology—Head and Neck Surgery by Daniel Ghods-Esfahani, Fatemeh Ramazani and Justin Lui in Journal of Otolaryngology - Head & Neck Surgery

sj-docx-5-ohn-10.1177_19160216261443996 – Supplemental material for Validating the Calgary Simulation Curriculum: A Retrospective Review of Face and Content Validity of a Surgical Simulation Curriculum in Otolaryngology—Head and Neck Surgery Supplemental material, sj-docx-5-ohn-10.1177_19160216261443996 for Validating the Calgary Simulation Curriculum: A Retrospective Review of Face and Content Validity of a Surgical Simulation Curriculum in Otolaryngology—Head and Neck Surgery by Daniel Ghods-Esfahani, Fatemeh Ramazani and Justin Lui in Journal of Otolaryngology - Head & Neck Surgery

sj-docx-6-ohn-10.1177_19160216261443996 – Supplemental material for Validating the Calgary Simulation Curriculum: A Retrospective Review of Face and Content Validity of a Surgical Simulation Curriculum in Otolaryngology—Head and Neck Surgery Supplemental material, sj-docx-6-ohn-10.1177_19160216261443996 for Validating the Calgary Simulation Curriculum: A Retrospective Review of Face and Content Validity of a Surgical Simulation Curriculum in Otolaryngology—Head and Neck Surgery by Daniel Ghods-Esfahani, Fatemeh Ramazani and Justin Lui in Journal of Otolaryngology - Head & Neck Surgery

sj-docx-7-ohn-10.1177_19160216261443996 – Supplemental material for Validating the Calgary Simulation Curriculum: A Retrospective Review of Face and Content Validity of a Surgical Simulation Curriculum in Otolaryngology—Head and Neck Surgery Supplemental material, sj-docx-7-ohn-10.1177_19160216261443996 for Validating the Calgary Simulation Curriculum: A Retrospective Review of Face and Content Validity of a Surgical Simulation Curriculum in Otolaryngology—Head and Neck Surgery by Daniel Ghods-Esfahani, Fatemeh Ramazani and Justin Lui in Journal of Otolaryngology - Head & Neck Surgery
