# Supplementary material for: Validating the Calgary Simulation Curriculum: A Retrospective Review of Face and Content Validity of a Surgical Simulation Curriculum in Otolaryngology—Head and Neck Surgery
Source: J Otolaryngol Head Neck Surg. 2026 Apr 27;55:19160216261443996. doi: 10.1177/19160216261443996 (PMC13133485; doi:10.1177/19160216261443996)
Supplement: sj-docx-1-ohn-10.1177_19160216261443996 – Supplemental material for Validating the Calgary Simulation Curriculum: A Retrospective Review of Face and Content Validity of a Surgical Simulation Curriculum in Otolaryngology—Head and Neck Surgery [file sj-docx-1-ohn-10.1177_19160216261443996.docx]

**SPA Ligation**

Fatemeh Ramazani, Justin Lui, Jessica Clark

**Objectives**

1. Understand the indications for performing surgical management of epistaxis.
2. Describe the relevant anatomy for performing SPA ligation.
3. Outline the procedural steps of SPA ligation.

**Background**

Trans-nasal endoscopic sphenopalatine artery ligation (TESPAL) is the most common surgical arterial ligation technique.

- Reported success rate of up to 98%.
- Low complication rates.
- Low rate of postoperative hemorrhage (3.4%).
- Similar reported mortality rate as compared with embolization.

**Indications for SPA Ligation**

1. Intractable posterior epistaxis, not stopped by adequate anterior and posterior nasal packing, with no clear source of bleed on examination.
2. Recurrent unilateral epistaxis unrelated to an underlying systemic disease.
3. Intra-operative management of hemorrhage during sinus surgery.
4. Devascularization of a vascular tumour (as an alternative to embolization).
5. Transpterygoid surgical approach.

**Contraindications for SPA Ligation**

1. Correctable coagulopathy.
2. Significant medical comorbidities, making the patient a poor candidate for general anaesthetic.

**Nasal Vascular Anatomy Review**

| **External Carotid Artery System** |
| --- |
| - Two major branches contributing to nasal vasculature   **Internal Maxillary Artery**   - - Lies more antero-inferior to vidian and maxillary nerves   - Enters the pterygomaxillary fossa and divides into:   - Sphenopalatine a.   - descending palatine a.   - pharyngeal a.   - infraorbital a.   - posterior superior alveolar a.   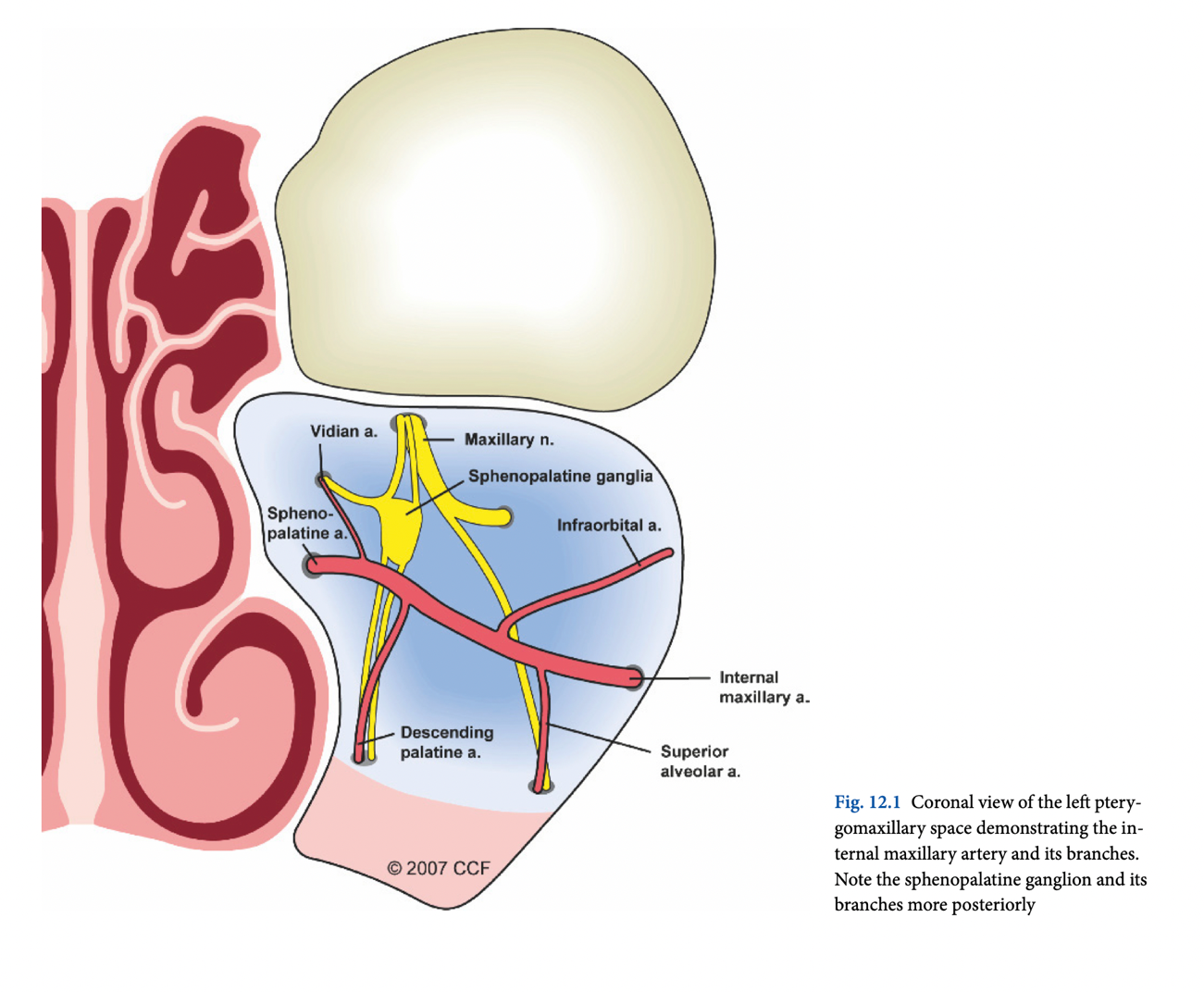  *Photo take from: Seth J. Kanowitz et al. Contemporary Management Strategies for Epistaxis.*  **Facial Artery**   - - Enters deep to platysma   - At the lip, splits into:     - Inferior labial artery     - Superior labial artery     - Angular artery   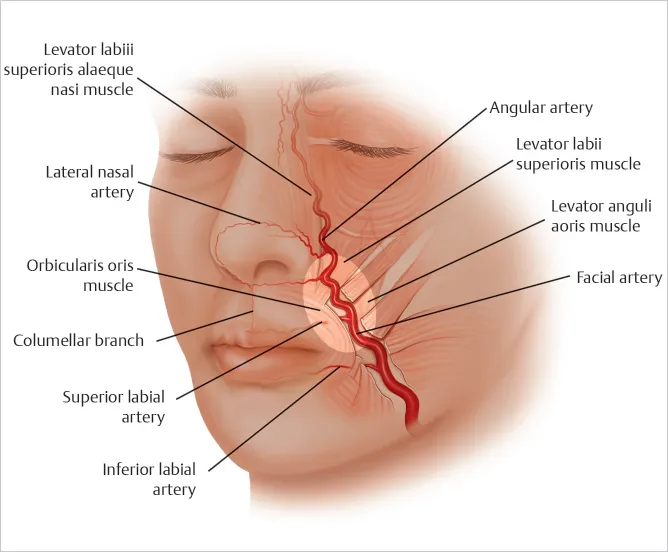  *Photo from: Plasticsurgerykey.com* |

| **Internal Carotid Artery System** |
| --- |
| - Two major branches contributing to nasal vasculature, both deriving from the ophthalmic artery and coursing through the ethmoid roof   - Anterior ethmoid a.   - Posterior ethmoid a.   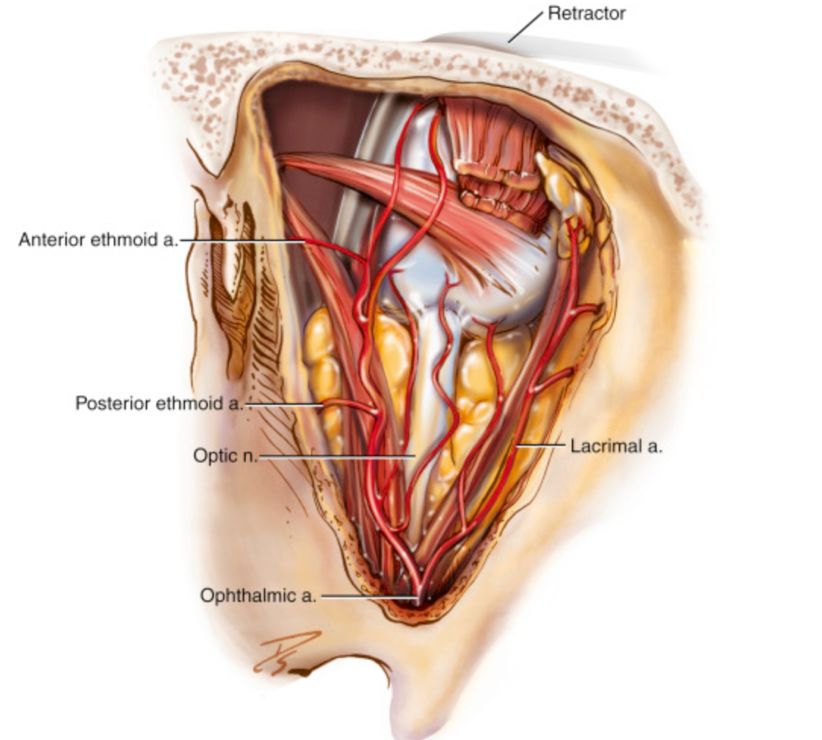  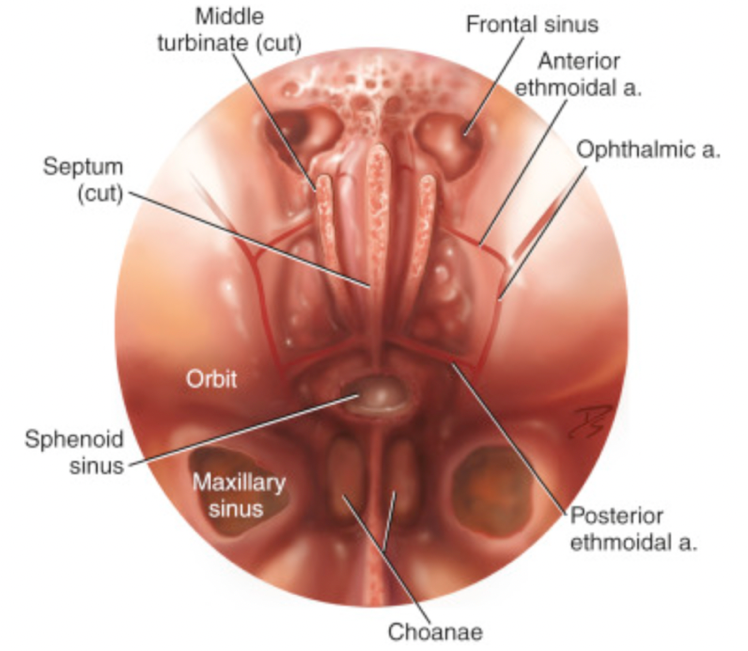 *Photos from:* [*Atlas of Endoscopic Sinus and Skull Base Surgery*](#!/browse/book/3-s2.0-C20150011935) *Second Edition.* |

| **The Sphenopalatine Artery** |
| --- |
| - SPA enters the nasal cavity through the sphenopalatine foramen, along the lateral nasal wall, in the region of the crista ethmoidalis   - Crista ethmoidalis is at the junction of:     - Inferior portion of the middle turbinate basal lamella     - medial orbital wall - Sphenopalatine foramen is located at the posterosu­perior corner of the maxillary sinus and deep to the posterior attachment of the middle turbinate   - Maxillary antrostomy can facilitate exposure of the SPA in this location   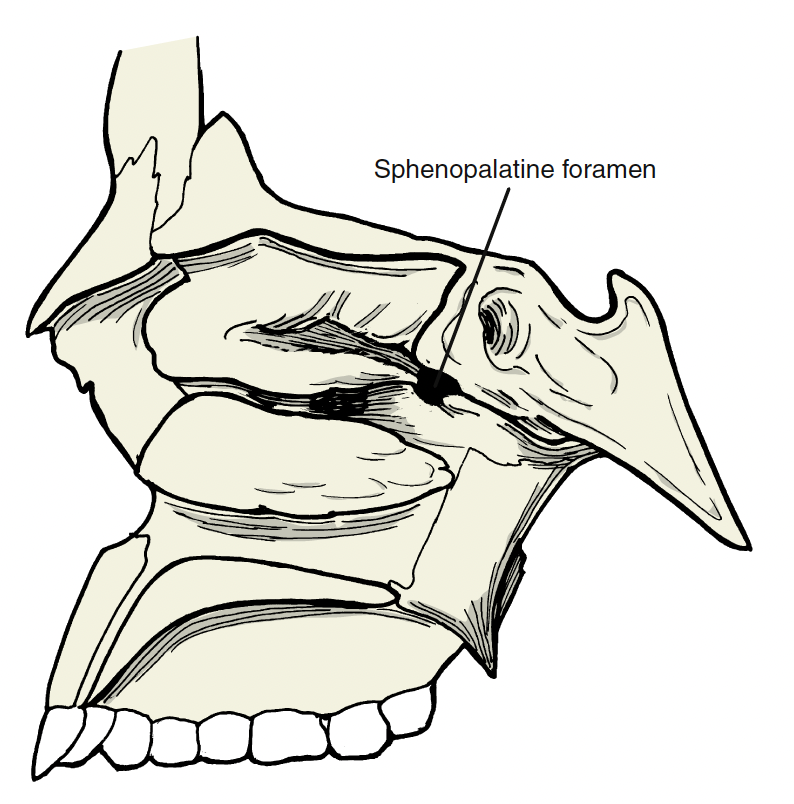  *Photo taken from: Snyderman & Pollack. Sphenopalatine Artery Ligation for Epistaxis.* |

**Operative Steps**

| **Surgical Field Preparation** | 1. Using a headlight and under direct visualization, place two nasal pledgets soaked in Epinephrine or Otrivin within the nasal cavity selected for SPA ligation. 2. Allow for adequate vasoconstrictive effect to take place (minimum 5 minutes). 3. Pack the oropharynx, in order to prevent accumulation of blood during surgery. |
| --- | --- |
| **Visualization of the Nasal cavity** | 1. Using a 0-degree rigid endoscope, examine bilateral nasal cavities, and the nasopharynx. 2. If visualization is impared due to bleeding, consider additional hemostasis with topical agents (otrivin or epinephrine) or suction cautery). |
| **Obtaining Access to the Middle Meatus** | 1. Medialize the middle turbinate using the Cottle elevator, to gain access to the middle meatus. Take care not to fracture the middle turbinate, as this can increase the risk for CSF leak at its attachment to the cribriform plate. 2. Consider septoplasty or resection of concha bullosa, if these anatomical variants are obstructive to the desired view. |
| **Local Anesthetic Injection** | 1. Using a spinal needle, inject the axilla of the middle turbinate with lidocaine and 1:100 000 epinephrine solution. |
| **Maxillary Antrostomy** | 1. Use the ball-tip-seeker to identify the uncinate process. 2. Perform uncinectomy using backbiting forceps. 3. Use the Pedi-90 to resect the superior aspect of the uncinate process. 4. Place the ball-tip-seeker within the natural os of the maxillary sinus, and widen the antrostomy by placing downward and backward pressure on the instrument. 5. Use the microdebrider to clean excess debris for better visualization. |
| **Identify the Sphenopalatine Foramen** | 1. Use the Cottle elevator to elevate the mucoperiosteum from the lateral nasal wall, after making a vertical incision ~1cm anterior to the posterior attachment of the middle turbinate. The incision should extend ~1cm inferiorly from the attachment of the middle turbinate.   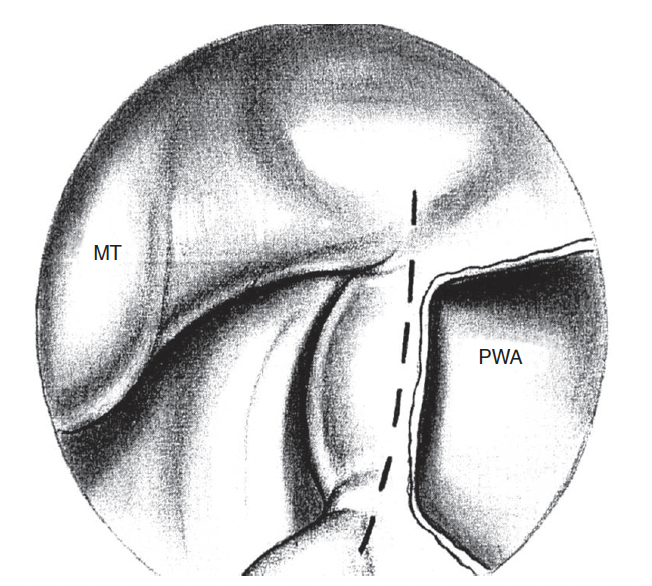  *Photo taken from: Snyderman & Pollack. Sphenopalatine Artery Ligation for Epistaxis.*   1. Dissect and list the mucoperiosteum posteriorly.   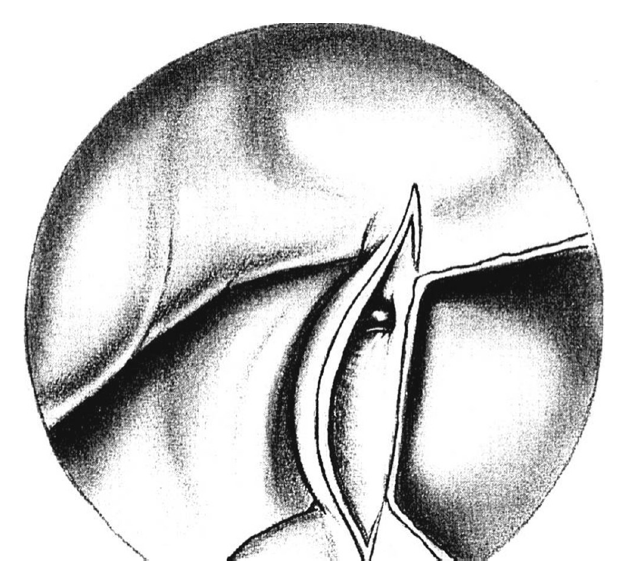  *Photo taken from: Snyderman & Pollack. Sphenopalatine Artery Ligation for Epistaxis.* |
| **Enlarge the Sphenopalatine Foramen** | 1. Use a Kerrison rongeur to place within the Sphenopalatine Foramen, superficial to the SPA. Remove the overlying bone with the rongeur and attempt to follow the SPA laterally into the pterygopalatine fossa.   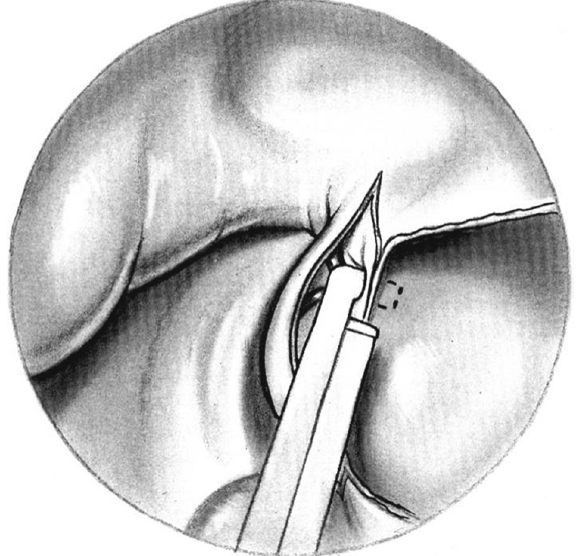  *Photo taken from: Snyderman & Pollack. Sphenopalatine Artery Ligation for Epistaxis.* |
| **Sphenopalatine Artery Ligation** | 1. Identify the SPA or its anterior branch and cauterize using bipolar or suction cautery.   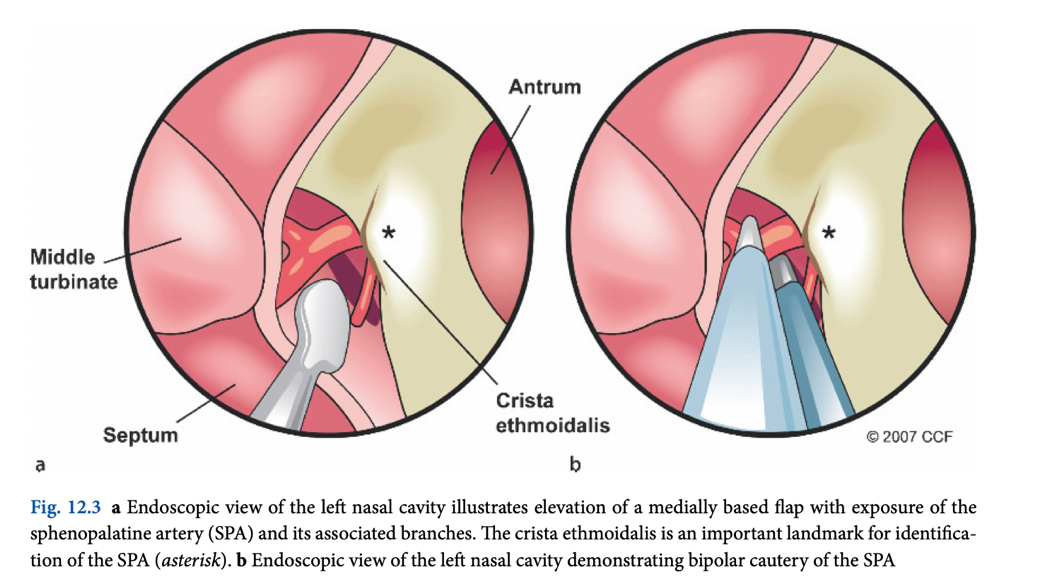  *Photo take from: Seth J. Kanowitz et al. Contemporary Management Strategies for Epistaxis.* |
| **Packing and Post-Procedure Care** | 1. Irrigate the nasal cavities with warm saline. 2. Identify and cauterize any other potential sources of bleeding. 3. Place absorbable nasal packing, or doyle splints if required. |
