# Supplementary material for: Validating the Calgary Simulation Curriculum: A Retrospective Review of Face and Content Validity of a Surgical Simulation Curriculum in Otolaryngology—Head and Neck Surgery
Source: J Otolaryngol Head Neck Surg. 2026 Apr 27;55:19160216261443996. doi: 10.1177/19160216261443996 (PMC13133485; doi:10.1177/19160216261443996)
Supplement: sj-docx-2-ohn-10.1177_19160216261443996 – Supplemental material for Validating the Calgary Simulation Curriculum: A Retrospective Review of Face and Content Validity of a Surgical Simulation Curriculum in Otolaryngology—Head and Neck Surgery [file sj-docx-2-ohn-10.1177_19160216261443996.docx]

**Nasal Fractures**

Fatemeh Ramazani, Justin Lui, Jessica Clark

**Objectives**

1. Understand the relevant nasal anatomy when managing a nasal fracture.
2. Identify the appropriate timeline for closed reduction of a nasal fracture.
3. List the indications for open reduction of a nasal fracture.
4. Outline the steps for performing regional nerve blocks for closed nasal reduction.
5. Outline the steps for performing close nasal reduction.

**Background**

- The nasal bones are the most commonly fractured facial bones, with blunt trauma being the most common cause.
- Untreated nasal fractures can result in cosmetic and functional (nasal breathing) deficits.
- Depending on the force of the trauma, the nasal bones can be laterally displaced or develop a comminuted fracture.


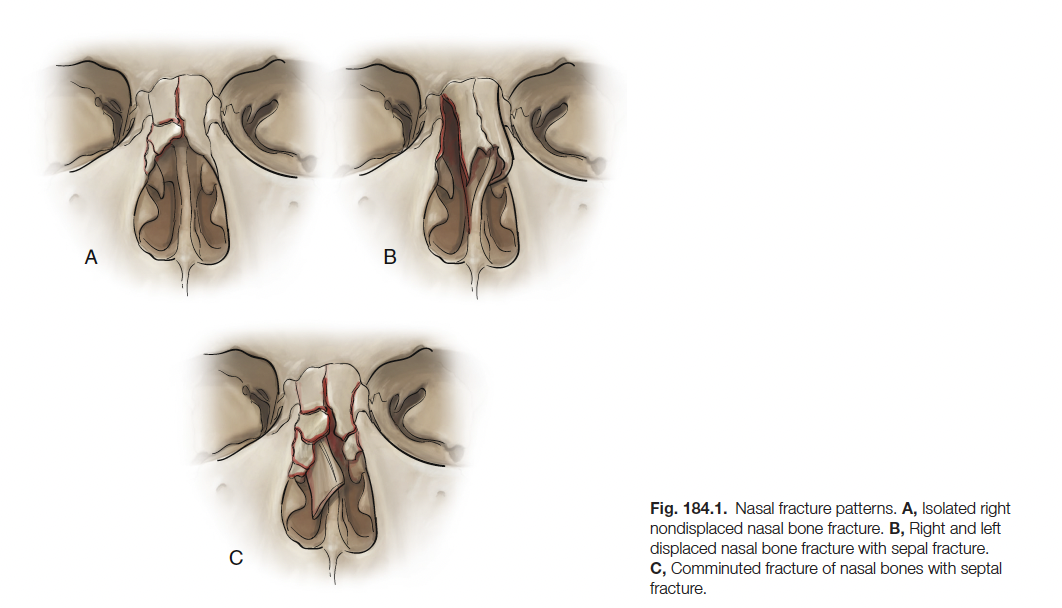


*Photo from: Rodriguez. Nasal Fracture.*

- Nasal fractures should be reduced within the first 24 hours of injury or 10-14 days after initial injury, if closed reduction is to be attempted.
  - Edema associated with the overlying soft tissue of the nose can distort the appearance, therefore giving an inaccurate representation of the deformity.
  - After 14 days, repair may require osteotomies.
- Closed reduction can be performed under GA or local anaesthetic.
- The nasal septum should be evaluated in all trauma cases, to rule out presence of a septal hematoma.

**Indications for Open Reduction**

1. Extensive fracture and/or dislocation of the nasal bones and septum
2. Nasal pyramid deviation greater than half the width of the nasal bridge
3. Fracture/dislocation of the caudal septum
4. Open septal fractures
5. Persistent deformity after closed reduction

**Review of Relevant anatomy**

| **Bony Anatomy** | Components of Nasal Septum   - Vomer - Perpendicular plate of ethmoid - Quadrangular cartilage - Anterior nasal spine - Palatine process of maxilla - Maxillary crest   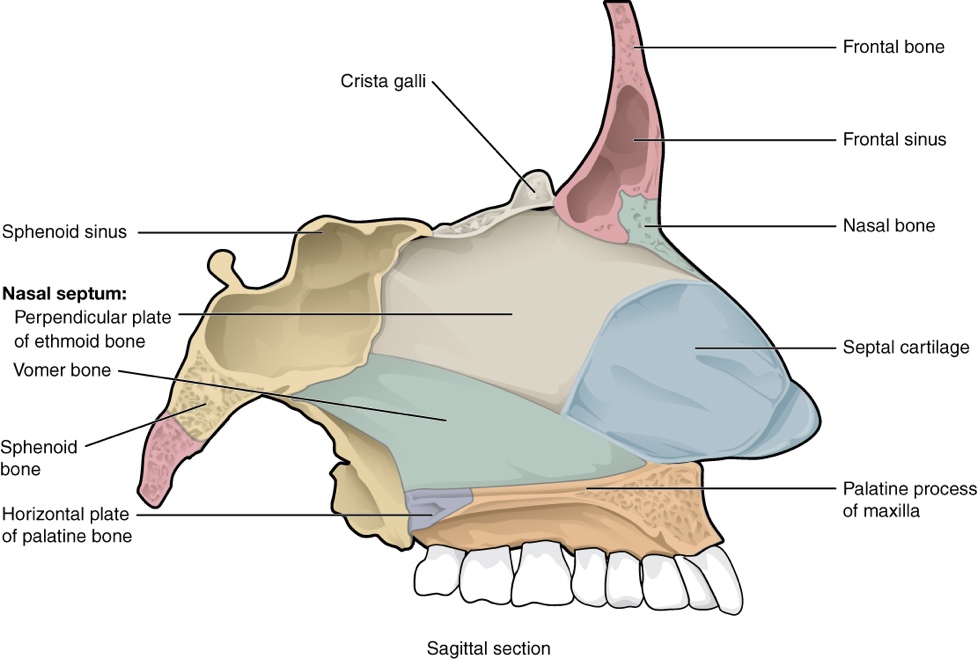  *Photo from: commons.wikimedia.org* |
| --- | --- |
| **Vascular Supply** | External   - Anteriorly: Angular artery (Facial artery) - Dorsally: Infraorbital and ophthalmic arteries   Internal   - Lateral nasal wall: Sphenopalatine artery (posteroinferiorly) - Superior: Anterior and posterior ethmoidal arteries |
| **Nasal Innervation** | Nasal sensory innervation come from branches of the trigeminal nerve:   1. Ophthalmic (V1) n.    1. Lacrimal n.    2. Frontal n.       1. Supratrochlear n. (supplies the root of the nose).    3. Nasociliary n.       1. Provides innervation to the septum and lateral nasal walls. Further divides into:          1. Posterior ethmoidal nerve.          2. Anterior ethmoidal nerve (anterior part of the septum, the lateral wall of the nasal cavity, the nasal bone, and skin to the tip of the nose).          3. Long ciliary nerves.          4. Communicating branches to the ciliary ganglion.          5. Infratrochlear nerve. 2. Maxillary (V2) n.    1. Nasopalatine n. Provides innervation to the septum and lateral walls    2. Infraorbital nerve supplies the wings of the nose and septum.     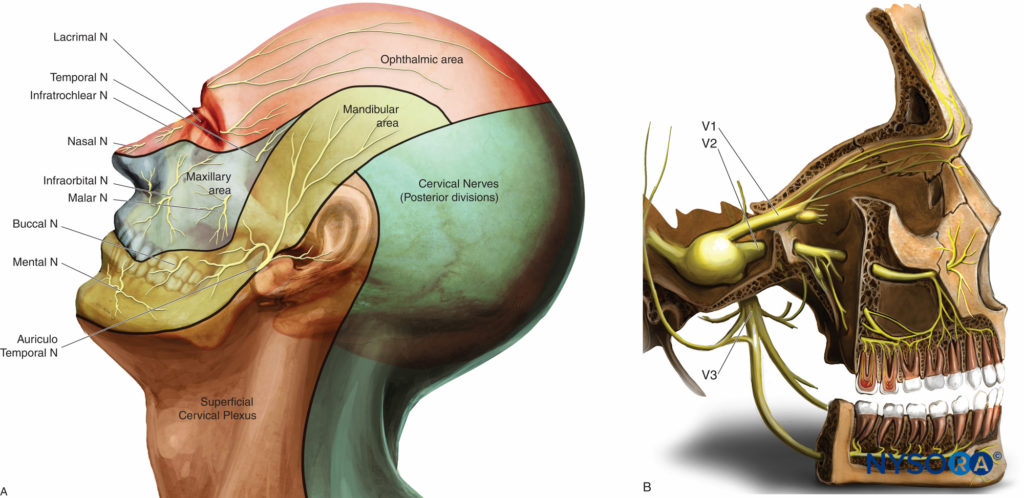  *Photo from: nysora.com* |

**Nerve Blocks**

| **General Principles** | - Anesthesia for closed reduction can be done under local +/- sedation, or under general anaesthetic. |
| --- | --- |
| **Topical Anaesthetic** | - Sensory nerves can be blocked at various locations:   - At their emergence point (V2 and V3).   - Distally and superficially, at their exit from the facial bones (V1, V2, V3). - Any nerve block should be accompanied by application of intra-nasal freezing, by applying nasal pledgets soaked in a cocktail of 4% lidocaine and 1: 100 000 epinephrine or otrivin (for vasoconstriction). |
| **Nasociliary nerve block** | - Blocked prior to its division into the ethmoidal infratrochlear nerves - Do not use epinephrine here, to avoid the risk of retinal artery spasm   **Steps:**   - Use a 25–27 gauge needle for injection. - Inject ~1cm above the medial canthus. - Direct the needle medially and backward to contact the bony roof of the orbit.   - At a depth of 1.5 cm, the needle should be at the anterior ethmoidal foramen. - Aspirate for blood and slowly inject. - Continue injective as you withdraw the needle, to provide local anaesthetic effect to the external nasal nerve. - Compress the interior angle of the eye with a finger to allow for diffusion of the anesthetic into the foramen.   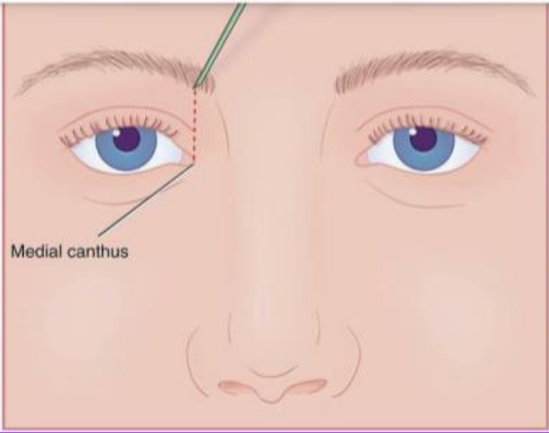  *Photo from: Aneskey.com* |
| **Infraorbital Nerve** | **Intraoral Approach**   - Palpate the infraorbital foramen - Below the orbital rim   - Can be identified by drawing a vertical line drawn through the center of the pupil and a horizontal line through the nasal ala - Palpate the incisor and the first premolar - Insert a 25-27 gauge needle into the buccal mucosa in the subsulcal groove at the level of the first premolar   - Direct the needle upward and outward into the canine fossa. - Keep a finger over the infraorbital foramen to assess the location of the needle tip to avoid damage to the orbit.   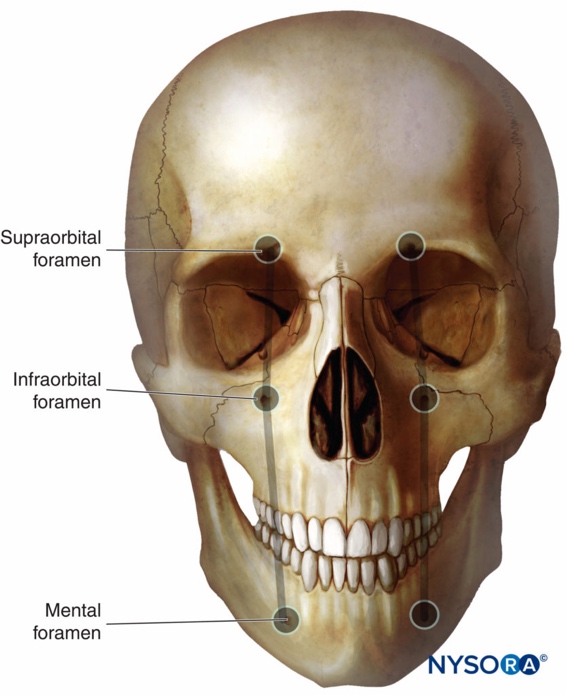  *Photo from: nysora.com*  **Extraoral Approach**   - Better for patient tolerance - Palpate the infraorbital foramen - Insert a 25-27 gauge needle perpendicularly with a cephalic and medial direction toward the foramen until bone is palpated - Keep a finger over the infraorbital foramen to assess the location of the needle tip to avoid damage of the orbit. |
| **Nasopalatine Nerve** | - Inject at the base of the columella and nasal floor |
| **Complications of Nerve Blocks** | - Palpebral edema - Diplopia - Transient paralysis of superior oblique - Ptosis - Ecchymosis at the puncture site - Hematoma |

**Operative Steps**

| **Pre-Operative Preparation** | Instruments commonly used for closed treatment of nasal fractures are:   - Headlight - Asch septum-straightening forceps - Walsham septum-straightening forceps - Boies nasal fracture elevator - Nasal speculum - Bayonet forceps - Nasal packing tray (be prepared to deal with resulting epistaxis)   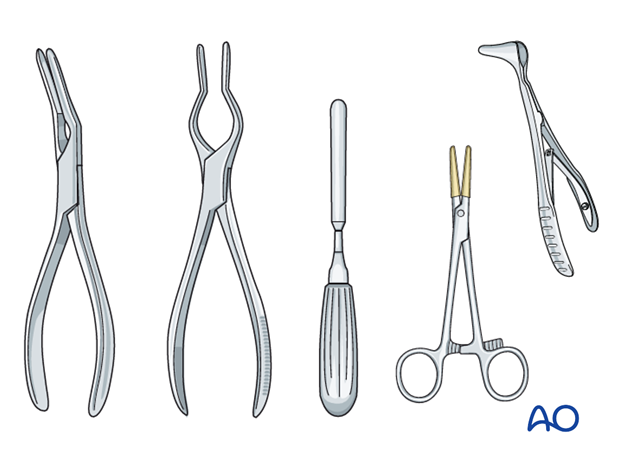  *Photo from: surgeryreference.aofoundation.org* |
| --- | --- |
| **Examination of the Nose** | - Examinate the extent of nasal deformity - Palpate the nasal bones to assess for:   - Mobility   - Step-offs with mid-face articulations   - Edema - Examine the nasal cavities using a nasal speculum and/or endoscope - Measure the distance between the superior aspect of the nasal bones and the external nasal valve (deepest point of entry with instruments).   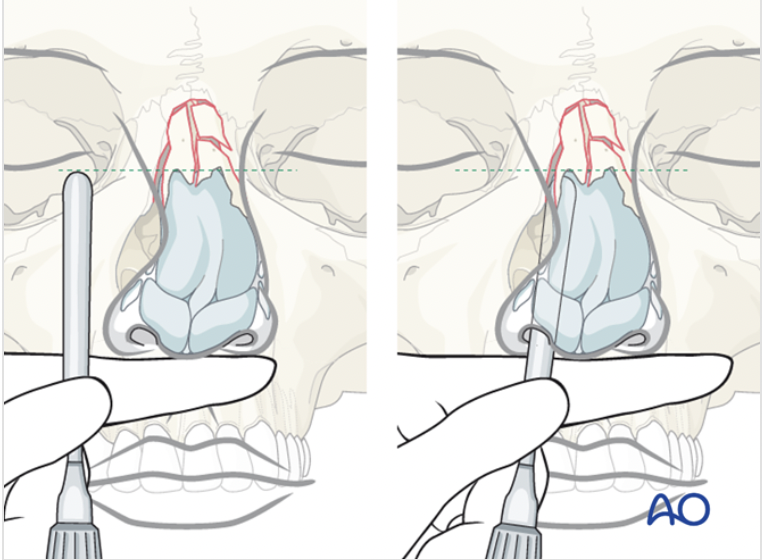  *Photo from: surgeryreference.aofoundation.org* |
| **Closed Reduction** | 1. Start with the side with greatest deformity. 2. Apply controlled pressure to the lateral nasal wall and with the Boies elevator, re-align the lateral nasal wall with controlled pressure.   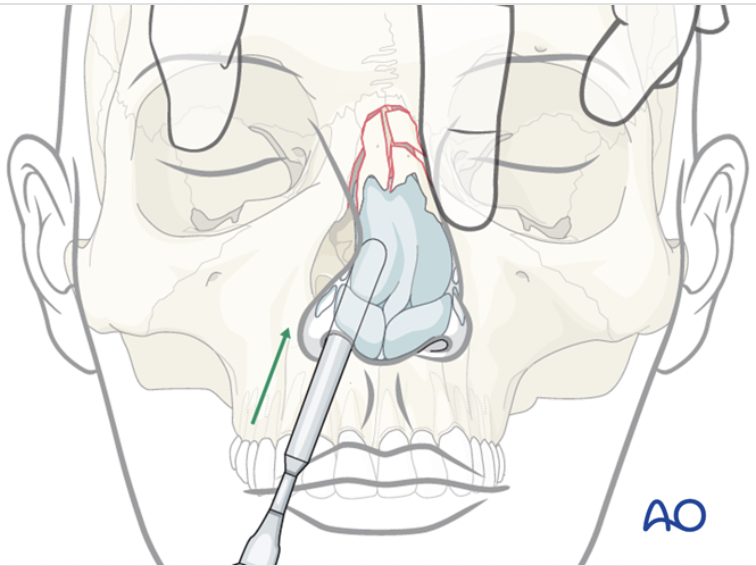  *Photo from: surgeryreference.aofoundation.org*   1. Palpate the nasal bones externally as they are being reduced internally, to provide tactile feedback on bony movement. 2. For management of septal deformities, place each prong of the Asch or Walsham straightening forceps and squeeze the handles together.   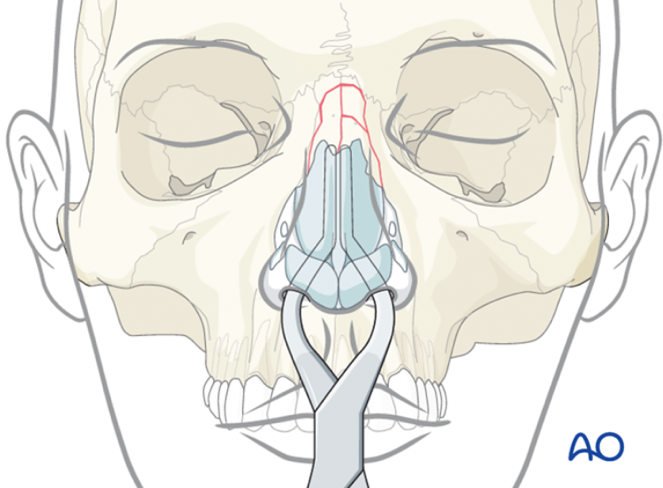  *Photo from: surgeryreference.aofoundation.org* |
| **Post-Operative Care** | - Manage any bleeding appropriately, if encountered pre- or post-reduction   - Consider nasal packing, if required - Place an external nasal split   - Place mastisol over the external nose and allow to dry   - Line steri-strips across the nose horizontally, and one across the columella   - Place the appropriately sized nasal cast over the steri-strips on the nose - Nasal split should be removed in 5-7 days   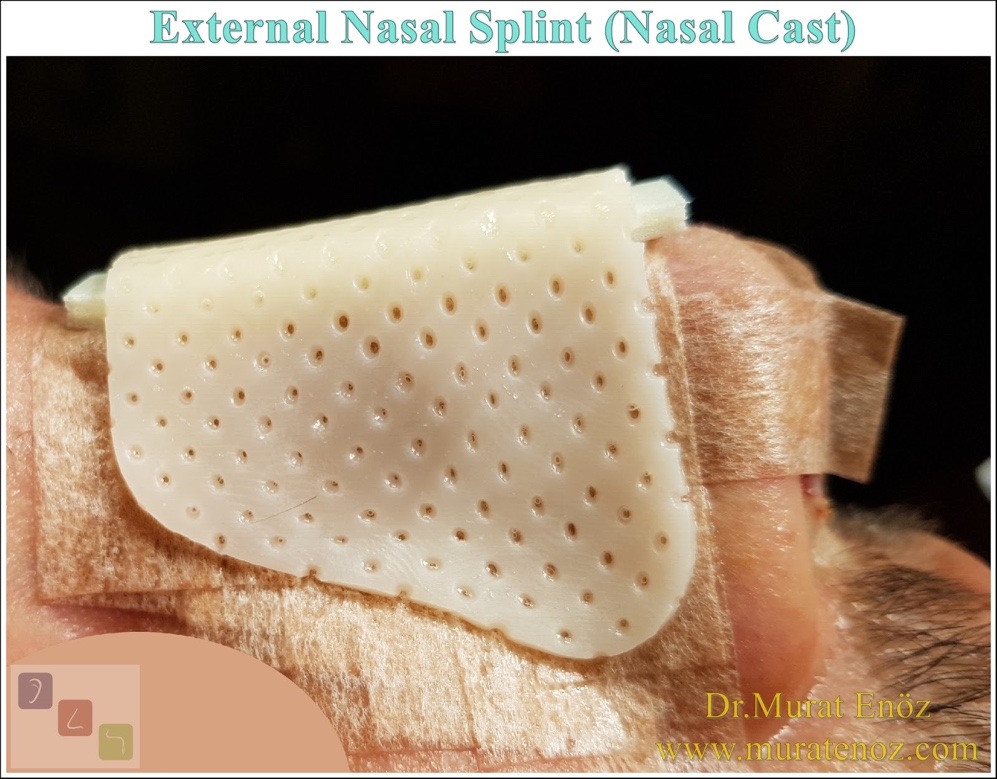  *Photo from: Entistanbul.com* |
