# Supplementary material for: Validating the Calgary Simulation Curriculum: A Retrospective Review of Face and Content Validity of a Surgical Simulation Curriculum in Otolaryngology—Head and Neck Surgery
Source: J Otolaryngol Head Neck Surg. 2026 Apr 27;55:19160216261443996. doi: 10.1177/19160216261443996 (PMC13133485; doi:10.1177/19160216261443996)
Supplement: sj-docx-3-ohn-10.1177_19160216261443996 – Supplemental material for Validating the Calgary Simulation Curriculum: A Retrospective Review of Face and Content Validity of a Surgical Simulation Curriculum in Otolaryngology—Head and Neck Surgery [file sj-docx-3-ohn-10.1177_19160216261443996.docx]

Resident Neck Dissection Manual

Fatemeh Ramazani, Justin Lui, Joseph Dort, Robert Hart, Wayne Matthews, Shamir Chandarana

**Objectives**

1. Understand the goals of performing a neck dissection for patients with head and neck cancer
2. List the pattern of nodal spread for the different subsites of head and neck cancer
3. Outline relevant history and physical examinations for patients presenting with head & neck malignancies, based on anatomical considerations (tongue paralysis, vocal fold paralysis, upper airway obstruction, etc.)
4. Perform the steps of a selective neck dissection

**Patient Presentations**

1. Oropharynx primary
   1. 4 Subsites:
      1. BOT (including lingual surface of epiglottis)
      2. Tonsil
      3. Posterior pharyngeal wall
      4. Oral surface of soft palate

| History | Physical Examination |
| --- | --- |
| **History of Present Illness:** Onset, Duration, Progression, Sidedness, Aggravating / Alleviating factors, Dysphagia, Odynophagia, Otalgia, Dyspnea, Globus, Hoarseness, dysarthria **Past Medical History:** Co-morbidities, Radiation exposure, previous H+N Cancer  **Family History**: Head and Neck Cancer  **Social**: Smoking, EtOH, Drug-use  **ROS:** Night sweats, fevers, chills, weight loss, current diet (able to swallow and eat?) | Complete Head and Neck Examination, including:   - General Assessment: Stridor, stertor, hemoptysis, difficulty managing secretions, muffled voice, dysphonia - Palpation of the neck for masses and lymphadenopathy - Oral cavity examination with a headlight and two tongue depressors   - Remove dentures - Palpation of the oropharynx as tolerated by the patient (particular attention to the tonsils/tonsillar fossa) - Otoscopy - Flexible nasopharyngoscopy, with specific maneuvers to assess the following sites:   - “Stick out your tongue” for the base of tongue   - “Puff out your cheeks” for the piriform sinuses   - Phonation for assessment of vocal cord mobility   - Chin to chest for assessment of subglottis |

1. Oral Cavity Primary
2. 7 Subsites
   1. Lip
   2. Alveolar/Gingival
   3. Buccal
   4. FOM
   5. Tongue
   6. RMT
   7. Hard Palate

| History | Physical Examination |
| --- | --- |
| **History of Present Illness:** Onset, Duration, Progression, Side, Aggravating / Alleviating factors, Dysphagia, Odynophagia, Otalgia, Dyspnea, Hoarseness, ill-fitting dentures, non-healing ulcers in the mouth, dysarthria  **Past Medical History:** Co-morbidities, Radiation exposure, previous H+N Cancer  **Family History**: Head and Neck Cancer  **Social**: Smoking, EtOH, Drug-use  **ROS:** Night sweats, fevers, chills, weight loss, current diet (able to swallow and eat?) | Complete Head and Neck Examination, including:   - General Assessment: Stridor, stertor, hemoptysis, difficulty managing secretions, muffled voice, dysphonia - Palpation of the neck for masses and lymphadenopathy - Oral cavity examination with a headlight and two tongue depressors   1. Remove dentures - Palpation of the oropharynx as tolerated by the patient (particular attention to the tonsils/tonsillar fossa) - Otoscopy - Flexible nasopharyngoscopy, with specific maneuvers to assess the following sites:   1. “Stick out your tongue” for the base of tongue   2. “Puff out your cheeks” for the piriform sinuses   3. Phonation for assessment of vocal cord mobility   4. Chin to chest for assessment of subglottis |

**Laryngeal SCC**

1. 3 Sites:
   1. Supra-Glottic
   2. Supra-Hyoid epiglottis --- Laryngeal surface
   3. Infra-Hyoid epiglottis
   4. Aryepiglottic fold
   5. False cords
   6. Arytenoid
   7. Glottic (Apex of ventricle to 1 cm below True VC)
   8. Subglottic (1 cm below True VC to Inferior border of Cricoid)

| History | Physical Examination |
| --- | --- |
| **History of Present Illness:** Onset, Duration, Progression, Aggravating / Alleviating factors, Dysphonia, Dyspnea, Dysphagia, Odynophagia, Otalgia, Hemoptysis, GERD  **Past Medical History:** Co-morbidities, Radiation exposure, previous H+N Cancer  **Family History**: Head and Neck Cancer  **Social**: Smoking, EtOH, Drug-use  **ROS:** Night sweats, fevers, chills, weight loss, current diet (able to swallow and eat?) | Complete Head and Neck Examination, including:   - General Assessment: Stridor, stertor, hemoptysis, difficulty managing secretions, muffled voice, dysphonia - Palpation of the neck for masses and lymphadenopathy - Oral cavity examination with a headlight and two tongue depressors   1. Remove dentures - Palpation of the oropharynx as tolerated by the patient (particular attention to the tonsils/tonsillar fossa) - Otoscopy - Flexible nasopharyngoscopy, with specific maneuvers to assess the following sites:   1. “Stick out your tongue” for the base of tongue   2. “Puff out your cheeks” for the piriform sinuses   3. Phonation for assessment of vocal cord mobility   4. Chin to chest for assessment of subglottis |

**Levels of the neck**

| Level | Subgroups | Boundaries | 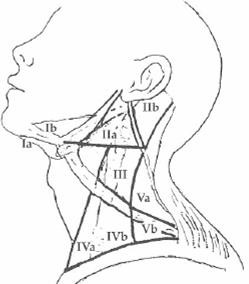 |
| --- | --- | --- | --- |
| IA | Submental | Between anterior digastric muscles and above the hyoid bone |  |
| IB | Submandibular | Above digastric (anterior and posterior bellies)  Below mandible |  |
| IIA | Upper Jugular | CN XI superiorly  Hyoid (clinical) or carotid bifurcation (surgical) inferiorly Anterior: Lateral border of Sternohyoid, posterior digastric, and stylohyoid  Posterior: Posterior border of SCM |  |
| IIB | Upper Jugular | Skull base superiorly  XI inferiorly |  |
| III | Middle Jugular | Superiorly carotid bifurcation (surgical) and inferiorly the intersection of omohyoid with IJV (surgical) OR hyoid to cricoid (clinical/radiological)  Anterior: Lateral border of sternohyoid  Posterior: Posterior border of SCM |  |
| IV | Lower Jugular | Clinical: Carotid to clavicle  Surgical: Intersection of omohyoid with IJV to clavicle Anterior: Lateral border of sternohyoid  Posterior: Posterior border of SCM |  |
| VA | Posterior triangle | From apex of level V to inferior border of cricoid cartilage Anterior: Posterior border of SCM  Posterior: Anterior border of the trapezius |  |
| VB | Posterior  triangle | From inferior border of cricoid to clavicle |  |
| VI | Central Compartment | Hyoid to suprasternal notch  Medial border of carotid sheaths bilaterally |  |

**Nodal Basins and Metastasis Patterns**

| Level | Subgroups | Pattern of Nodal Metastasis |
| --- | --- | --- |
| IA | Submental | Chin Lower Lip  Anterior FOM Tip of Tongue  Anterior mandibular alveolus/gingiva |
| IB | Submandibular | Oral Cavity (FOM, Oral Tongue) Nasal cavity (anterior)  Lower face  **Lower face and anterior nasal cavity are more likely to spread to the perifacial nodes at or above  border of the mandible |
| IIA | Upper Jugular | Oral cavity Nasal cavity Nasopharynx Oropharynx Hypopharynx Larynx Parotid  Skin |
| IIB | Upper Jugular | Oropharynx>oral cavity or larynx primary Parotid  Skin (especially of ear, temporoparietal scalp)  **inclusion in N0 neck is controversial |
| III | Middle Jugular | Oral cavity Nasopharynx Oropharynx Hypopharynx  Larynx |
| IV | Lower Jugular | Oropharynx  Hypopharynx |

|  |  | Larynx Thyroid  Cervical Esophagus |
| --- | --- | --- |
| VA | Posterior triangle | Nasopharynx Oropharynx  Posterior maxilla/paranasal sinuses  Skin and scalp |
| VB | Posterior triangle | Nasopharynx Oropharynx  Posterior maxilla/paranasal sinuses Skin and scalp |
| VI | Central Compartment | Thyroid  Larynx (glottic, subglottic) Hypopharynx (apex. piriform)  Cervical esophagus |

**Dissection Manual**

| **Incision** | 1. Pertinent Anatomy for incision marking:    1. Angle of the mandible,    2. Mastoid tip,    3. Midline of neck,    4. Sternal notch. 2. The Apron incision (from mastoid tip into a transverse lower neck skin crease) is typically used. 3. Be mindful of the proximity of your incision to the tracheostomy site. |
| --- | --- |
| **Skin Flap Elevation** | 1. Standard subplatysmal flap elevation. 2. Attempts at preserving the great auricular nerve should be made. 3. The external jugular vein should be preserved for reconstructive purposes. 4. If a tracheotomy has been performed, every attempt is made to keep the neck dissection separate from the tracheotomy site. 5. Boundaries of flap elevation:    1. Superior limit: mandible, mastoid tip, and parotid gland    2. Inferior limit: clavicle    3. Anterior limit: sternohyoid muscle    4. Posterior limit: SCM |
| **Submandibular and**  **submental dissection (Level** | 1. Marginal mandibular branch of the facial nerve is identified |

| **I), with dissection of perifacial lymph nodes** | 1. Runs below the angle of the mandible proximal and superficial to the posterior facial vein. The nerve may fall lower than expected in the elderly patient. 2. Lies within the submandibular fascia before turning superiorly to the lower lip. 3. Once identified, the nerve is carefully elevated off the underlying soft tissue and removed out of the plane of dissection. 4. The facial vessels are transected as they cross the mandible. 5. The contralateral anterior belly of the digastric muscle is skeletonized. 6. The superficial fat (submental fat) over the anterior bellies of both digastric muscles is dissected. 7. The dissection is continued over the ipsilateral mylohyoid muscle, which is then retracted superiorly by an assistant, while the submandibular gland is retracted inferiorly. 8. The lingual nerve and submandibular ganglion are identified by retraction of the mylohyoid muscle. 9. The submandibular ganglion and duct are identified, divided, and ligated (with a tie, clip, or harmonic). 10. The contents of the submandibular triangle are dissected, and the gland is delivered downward.     1. The facial artery is divided a second time at the posterior aspect of the gland,   preserving length on the facial artery for reconstruction if necessary |
| --- | --- |

|  | 9. The specimen is rolled off the posterior belly of the digastric muscle, by skeletonizing the posterior belly of the digastric back to the SCM. |
| --- | --- |
| **Jugular chain dissection (Levels II-IV)** | 1. The investing fascia of the SCM is divided along its length (“unzipping the SCM”).    1. Position an assistant to retract the medial aspect of the SCM with Allis clamps or manual pressure. Apply counter-traction on the muscle using a sponge in one hand while dissecting with the other. 2. The spinal accessory nerve is identified and traced from the SCM to the digastric muscle and skeletonized from the surrounding soft tissue. 3. level IIB: dissect the fatty tissue deep to the superior portion of the SCM, overlying the deep neck musculature. The spinal accessory nerve must be skeletonized all around, such that level IIB may pass under the nerve and remain in continuity with the specimen. 4. Identify the lateral border of the IJV at both the superior and inferior most aspects of the neck dissection.    1. The omohyoid muscle may need to be cut for level IV exposure. 5. Identify the phrenic nerve at the inferior aspect of the dissection using blunt dissection pushing with dental rolls.    1. The anterior scalene muscle can be used to assess the depth for finding the phrenic nerve, which lies superficial to the muscle in the posterior triangle of the neck. 6. Come across the floor of the neck by skeletonizing the cervical rootlets and dissecting the fat   and fascia anteriorly toward the internal jugular vein. |

|  | a. Ensure preservation of the cervical rootlets (preserving sensory to neck), brachial plexus, and phrenic nerve when doing this dissection.   1. The contents of the neck dissection should be carried from the back of the SCM to the carotid sheath. 2. Incise into the carotid sheath to unwarp the IJV. 3. Next, the specimen is sharply divided off the internal jugular vein just superficial to the adventitia (“white lining the jugular”).    1. Make sure to stay on the anterior border of the IJV. 4. To establish the anterior boundary of the neck dissection, reflect and dissect the contents superiorly along the undersurface of the omohyoid muscle (if omohyoid was transected inferiorly). |
| --- | --- |
| **Closure** | Skin flaps are closed in 2 layers:   1. Platysma/subcutaneous layer with 3-0 Vicryl, and 2. Skin with 4-0 Monocryl in a running subcuticular fashion. |
