# Supplementary material for: Validating the Calgary Simulation Curriculum: A Retrospective Review of Face and Content Validity of a Surgical Simulation Curriculum in Otolaryngology—Head and Neck Surgery
Source: J Otolaryngol Head Neck Surg. 2026 Apr 27;55:19160216261443996. doi: 10.1177/19160216261443996 (PMC13133485; doi:10.1177/19160216261443996)
Supplement: sj-docx-4-ohn-10.1177_19160216261443996 – Supplemental material for Validating the Calgary Simulation Curriculum: A Retrospective Review of Face and Content Validity of a Surgical Simulation Curriculum in Otolaryngology—Head and Neck Surgery [file sj-docx-4-ohn-10.1177_19160216261443996.docx]

# Resident Pediatric Airway Simulation Manual

Kiana Mahboubi, Fatemeh Ramazani, Justin Lui, Jessica Clark, Derrick Drummond, James Brookes

## Objectives

1. Identify key history and physical examination features of a pediatric foreign body aspiration presentation.
2. Understand the radiographic findings of pediatric foreign body aspiration.
3. Assess indications for immediate versus delayed foreign body retrieval.
4. Create an appropriate set up of operating room equipment (laryngoscope, bronchoscope, and forceps).
5. Effectively communicate with anesthesia, respiratory therapy, and nursing.
6. Develop a comprehensive post-operative plan.

**Patient Presentations**

| **Acute** | |
| --- | --- |
| **History** | **Physical Examination** |
| **History of Present Illness:** Onset, Duration, Progression, Aggravating / Alleviating factors, Dysphagia, Odynophagia, Dyspnea, Hoarseness, coughing, choking, vomiting, witnessed/unwitnessed, type of foreign body  **Past Medical History:** previous intubation, previous foreign body aspiration, term/preterm, syndromes **Family History**: bleeding disorders  **Social**: Socioeconomic status  **ROS:** NPO status, fever | Complete Head and Neck Examination, including:   - General Assessment: Vitals, Stridor (inspiratory, biphasic, expiratory), stertor, hemoptysis, difficulty managing secretions, muffled voice, dysphonia - ***if patient unstable (tripoding/drooling/low O2) call for help, avoid agitation, may have to secure airway immediately *** - Palpation of the neck for masses and lymphadenopathy - Oral cavity examination with a headlight and two tongue depressors - Chest auscultation: decreased air entry, wheeze, adventitious sounds |

| **Subacute** | |
| --- | --- |
| **History** | **Physical Examination** |
| **History of Present Illness:** Onset, Duration, Progression, Aggravating / Alleviating factors, Dysphagia, Odynophagia, Dyspnea, Hoarseness, coughing, choking, vomiting, witnessed/unwitnessed, type of foreign body  **Past Medical History:** previous intubation, previous foreign body aspiration, term/preterm, syndromes **Family History**: bleeding disorders  **Social**: Socioeconomic status  **ROS:** NPO status, fever, pneumonia, hemoptysis | Complete Head and Neck Examination, including:   - General Assessment: Vitals, Stridor (inspiratory, biphasic, expiratory), stertor, hemoptysis, difficulty managing secretions, muffled voice, dysphonia - ***if patient unstable (tripoding/drooling/low O2) call for help, avoid agitation, may have to secure airway immediately *** - Palpation of the neck for masses and lymphadenopathy - Oral cavity examination with a headlight and two tongue depressors - Chest auscultation: decreased air entry, wheeze, adventitious sounds |

| **Radiographic Findings** | |
| --- | --- |
| - Modality of choice is CXR   - Both inspiratory and expiratory views - Look for:   - Air trapping   - Hyperinflation   - Mediastinal shift   - Atelectasis   - Increased radiolucency | 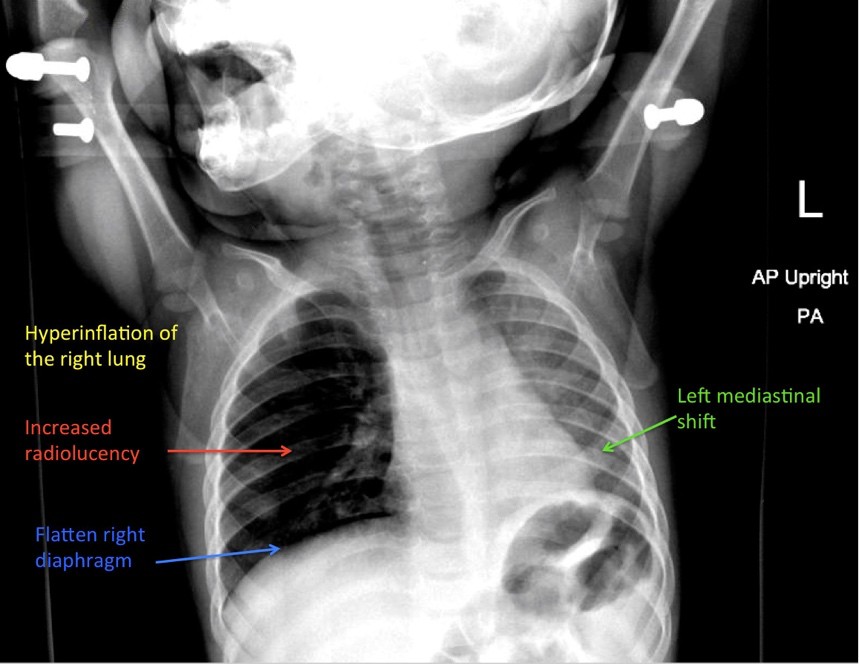 |

| **Valve mechanisms of airway obstruction with FBs** | | | | |
| --- | --- | --- | --- | --- |
| **Mechanism** | **Check Valve** | **Bypass Valve** | **Stop Valve** | **Ball Valve** |
| **Outline** | Air can get in but not out. | Partial obstruction only, resulting in reduced airflow on inspiration and expiration. | Complete obstruction, with no air moving in or out. | Air can get out but not in (Rare). |
| **CXR**  **Findings** | Hyperinflation on expiratory view, mediastinal shift to opposite side. | Normal | Collapse of affected segment | Atelectasis |

**Immediate VS Delayed FB Retrieval**

# Pediatric Patient with Airway Foreign Body

**Immediate** Complete obstruction Patient decompensating

## Delayed

Subacute organic matter that have caused ++ inflammation and edema and patient would benefit from a period of antibiotics and steroids to decrease the inflammation

## Procedure set-up

1. **Consent**
   - Discuss risks of
     - Anesthetic
     - Dental/laryngeal injury
     - FB impaction (worse obstruction)
     - Mucosal tear (bleeding/perf)
     - Pneumothorax
     - Inability to extract (need for further surgery)
     - Airway edema (leading to obstruction)
     - Post-obstruction pulmonary edema
     - Pneumonia
     - Granulation tissue
     - Stricture

## Team

- - Anesthesia and RT
    - Key Points for communication:
      - Who will spray the vocal cords with Lidocaine?
      - Method of ventilation
      - When you are taking over the airway
      - Your view of the larynx (Grades 1-4)
  - Nursing
    - Ensure that your nursing staff understands your setup and know which instruments they should be handing to you
  - ENT
  - Consider ICU if patient unstable or has significant airway edema

## Positioning

- - Shoulder roll
  - No donut (allows for head movement during procedure)
  - Bed placement (90 degrees head of bed turn from anesthesia, to allow for easy exchange of airway control between ENT and anesthesia)

## Laryngoscope

- - Parsons
  - Anesthesia MAC blade (preferred based on availability and removing the laryngoscope easily once the bronchoscope is past the cords)

## Bronchoscope

- - Size
  - Set up (Accessories)
  - Back up (one size smaller)
  - Light source
  - Camera
  - Suction

## Endotracheal tubes

- - Different sizes available for
    - Intermittent intubation/ventilation
    - Leaving patient intubated if significant edema/not safe to extubate

| **Rigid Bronchoscopy for Dummies** | |
| --- | --- |
| Pick the Right Size | 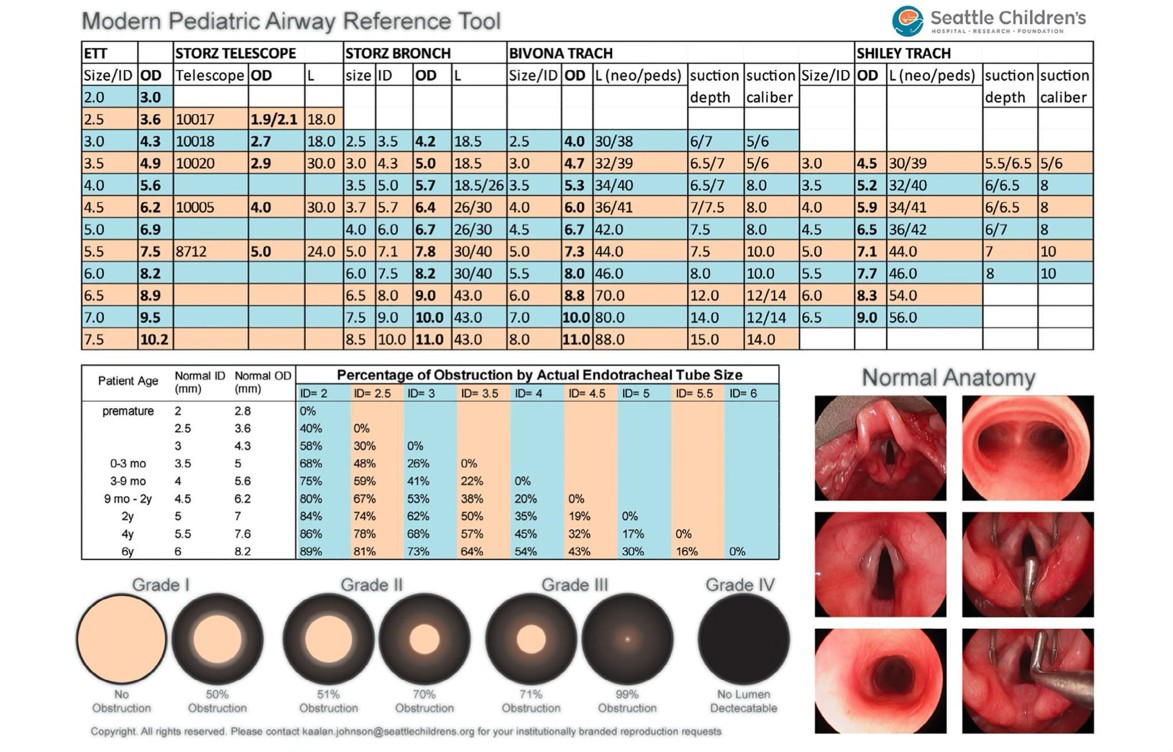 |
| Prismatic Light Deflector (Allows for passage of light through the scope) | 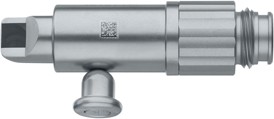 |

| Rubber Telescope Guide (Can help position the forceps within the bronchoscope) | 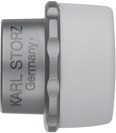 |
| --- | --- |
| Guide Piece Suction Catheter (Guides flexible suction through the side of a rigid bronchoscope) | 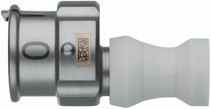 |
| Telescope Bridge (Helps achieve length compatibility between operative forceps, camera, and bronchoscope) | 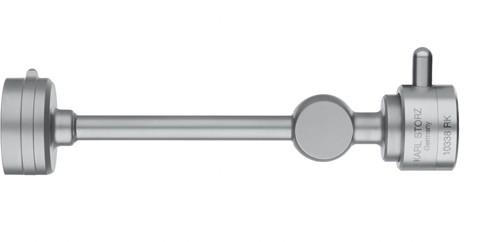 |
| Optical Forceps (Gives you the ability to operate and directly visualize) | 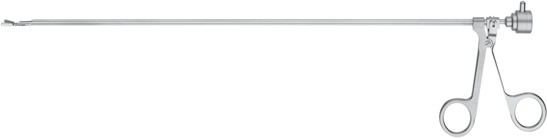 |
| Peanut Grasper | 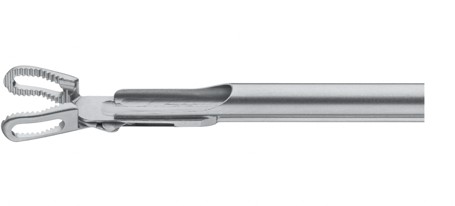 |
| Peanut Grasper for Peanut Fragments | 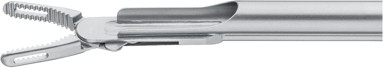 |

| **What am I looking at? Bronchoscope views of the lung:** | 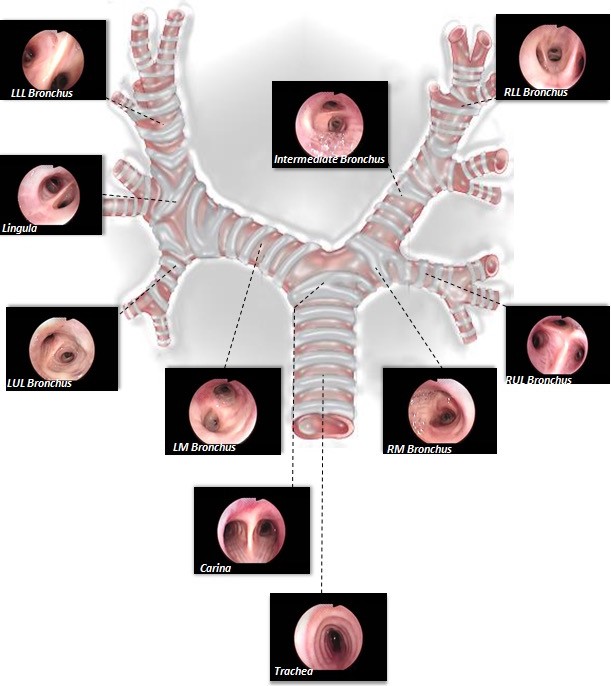 |
| --- | --- |

| **Intra-operative Troubleshooting and Post-Operative Planning** | |
| --- | --- |
| **Scenario** | **Plan** |
| **Foreign body retrieved but you lose grip of it in the trachea. What do you do?** | Push back in the previously obstructed bronchus, to avoid worsening obstruction from tracheal obstruction |
| **Significant tracheal or bronchial edema. You are therefore unable to visualize or grab foreign body. What do you do?** | Consider keeping patient intubated and sending to the ICU with steroids, and re-assessing with repeat bronchoscopy in 24 hours |
| **Foreign body removed successfully. What do you do next?** | Re-assess the airway for a second foreign body (5% of patients will have a second foreign body) |
| **Post-operative planning** | - Most patients can be discharged on the same day if stable - Steroids: 0.5 mg/kg x 3 doses and nebulized epinephrine if worried about airway edema - Consider antibiotics - Parental education on airway FB |
