# Supplementary material for: Validating the Calgary Simulation Curriculum: A Retrospective Review of Face and Content Validity of a Surgical Simulation Curriculum in Otolaryngology—Head and Neck Surgery
Source: J Otolaryngol Head Neck Surg. 2026 Apr 27;55:19160216261443996. doi: 10.1177/19160216261443996 (PMC13133485; doi:10.1177/19160216261443996)
Supplement: sj-docx-5-ohn-10.1177_19160216261443996 – Supplemental material for Validating the Calgary Simulation Curriculum: A Retrospective Review of Face and Content Validity of a Surgical Simulation Curriculum in Otolaryngology—Head and Neck Surgery [file sj-docx-5-ohn-10.1177_19160216261443996.docx]

Functional Endoscopic Sinus Surgery Dissection Manual

August 8^th^, 2024

Objectives:

1. Review indications for FESS
2. Describe the paranasal sinus anatomy and FESS landmarks
3. Understand the basic FESS instruments and equipment set up
4. Understand the basic steps of FESS
   1. Uncinectomy
   2. Maxillary antrostomy
   3. Anterior and posterior ethmoidectomy
   4. Sphenoidotomy
   5. Frontal sinuses

Videos that maybe helpful:

<https://www.youtube.com/watch?v=dLBHxltL238>

<https://www.youtube.com/watch?v=cIBnAWFr5yY>

**1.0: Indications for FESS**

- Chronic rhinosinusitis (+/- nasal polyposis)
- Recurrent acute rhinosinusitis
- Complications of rhinosinusitis (ex. Orbital cellulitis/abscess, cavernous sinus thrombosis)
- Sinonasal mycetoma
- Eosinophilic fungal rhinosinusitis
- Invasive fungal rhinosinusitis
- Sinonasal tumours (ex. Inverted papilloma, mucoceles)

**2.0: Goals of “Functional” Endoscopic Sinus Surgery**

1. Enlarge sinus ostia
2. Restore adequate aeration of sinuses
3. Provide a better route for topical therapies
4. Maintain the natural mucociliary clearance

**3.0: Review of Paranasal Sinus Anatomy & landmark**

| Four Consistent Landmarks for FESS:   1. Uncinate Process (1) 2. Anterior wall of Ethmoid Bulla (2) 3. Basal lamella of the Middle Turbinate (3) 4. Face of the Sphenoid (4)   Three Inconsistent Landmarks for FESS:   1. Posterior wall of Ethmoid Bulla (2a) 2. Superior Turbinate Basal Lamella (3a) 3. Supreme Turbinate Basal Lamella (3b) | 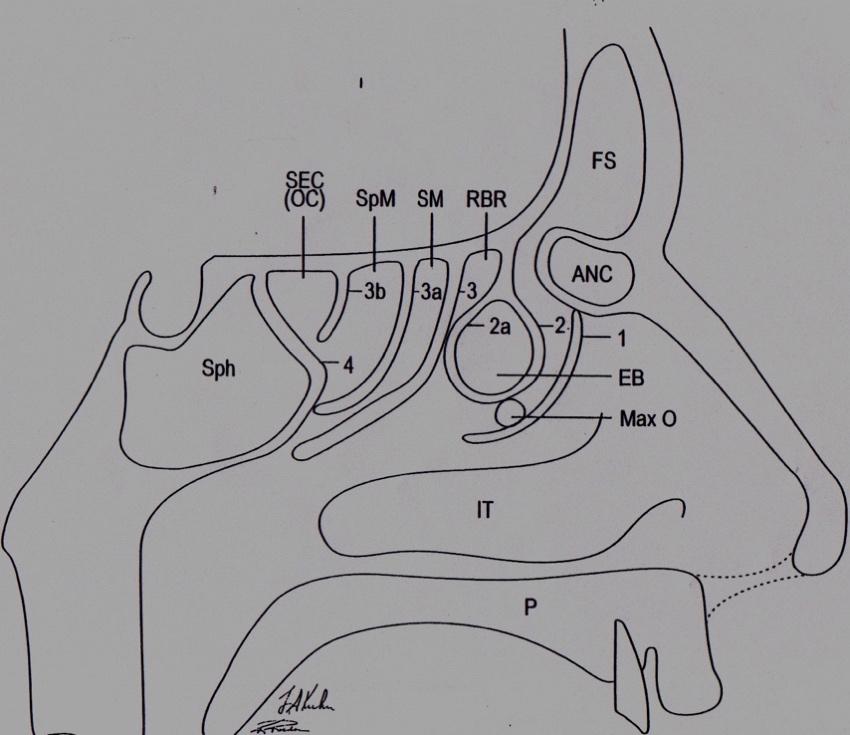 |
| --- | --- |
| Osteomeatal Complex:  The final common drainage pathway for the frontal, maxillary and anterior ethmoid cells.  Includes 5 structures: maxillary sinus ostium, anterior ethmoid cells, infundibulum, hiatus semilunaris, middle meatus (passage between the middle turbinate and the lateral nasal wall / inferior turbinate). |  |
| Uncinate Process   1. Type A: Lamina Papyracea  (formation of a recessus terminalis – red arrow) 2. Type B: Roof of Ethmoid 3. Type C: Middle Turbinate | 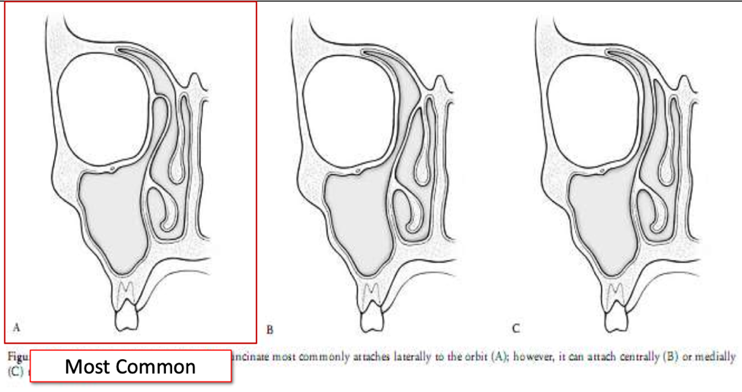 |
| Middle Turbinate   1. Anterior Buttress 2. Vertical attachment 3. Posterior attachment to medial orbital wall (Basal Lamella) – separates anterior from posterior ethmoid 4. Posterior Buttress (attaches to lateral nasal wall) | 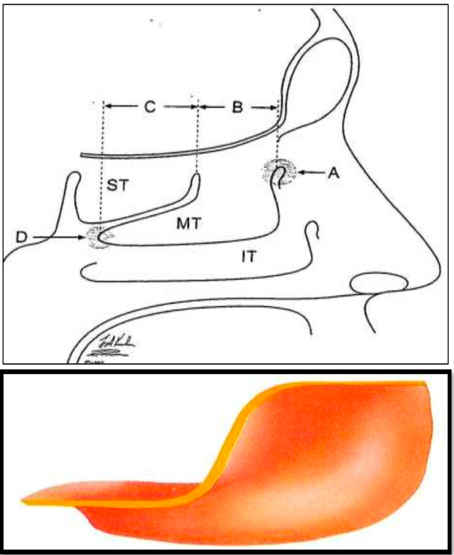 |
| Hiatus Semilunaris:   - H. Semilunaris inferioris: space between free posterior margin of uncinate process and anterior face of ethmoid bulla - H. Semilunaris superioris: space between ethmoid bulla and middle turbinate | 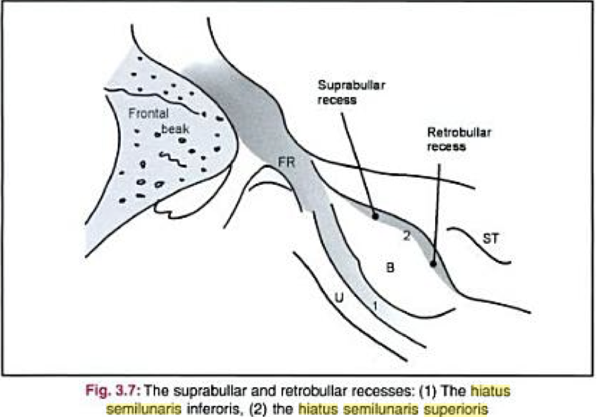 |
| Infundibulum:   - Funnel shaped space bounded by the uncinate medially and lamina papyracea laterally. - opens into middle meatus via hiatus semilunaris inferioris. | 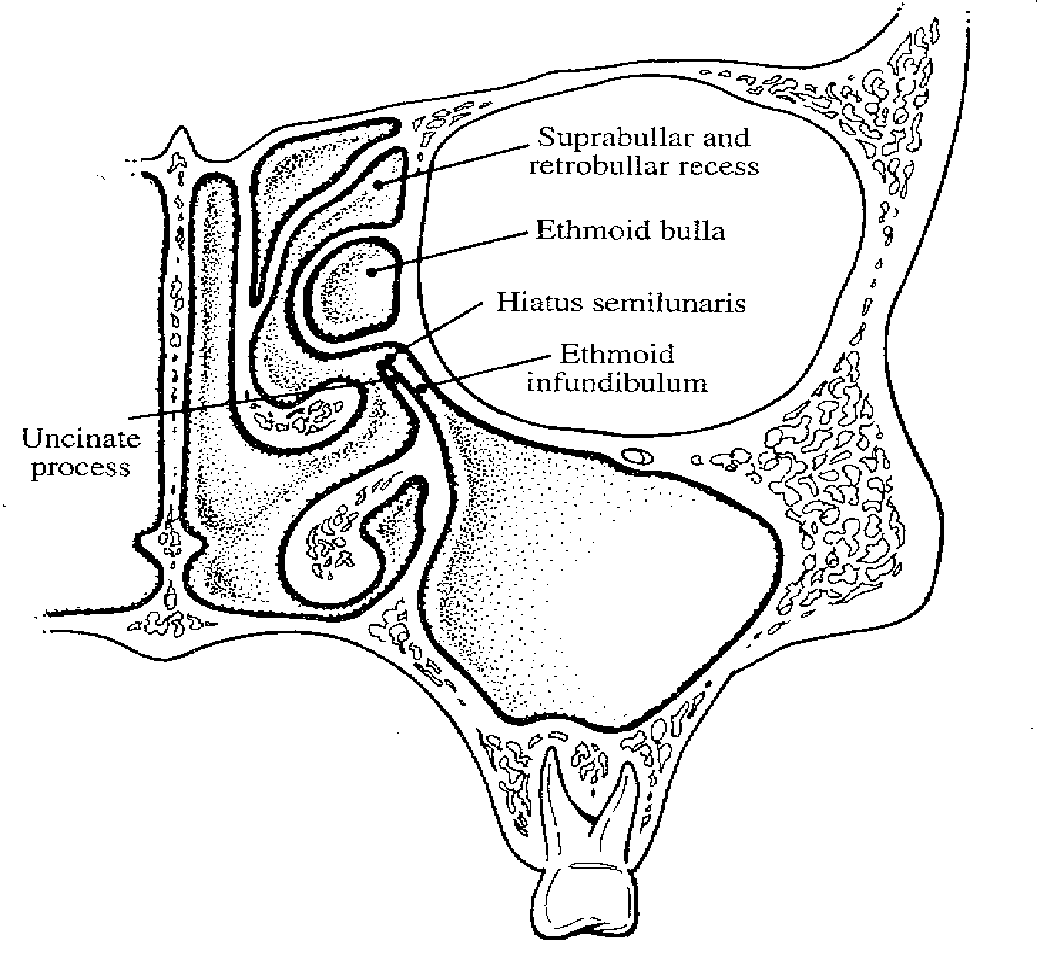 |
| Ethmoid roof configurations: Keros classification   1. Type I: 1-3mm deep 2. Type II: 4-7mm deep 3. Type III: 8-16mm deep 4. Type IV: Asymmetrical | 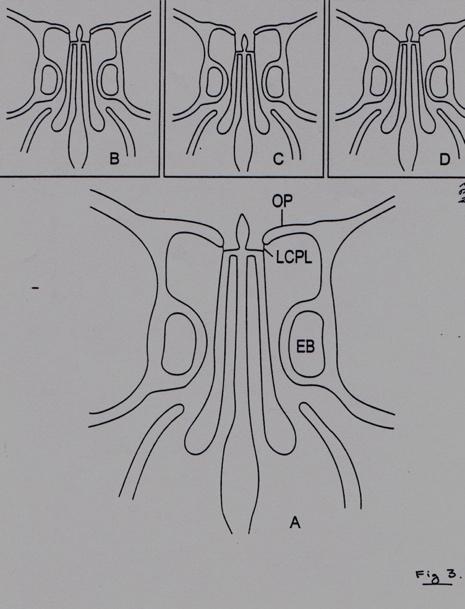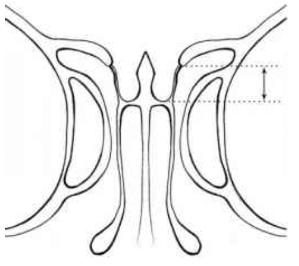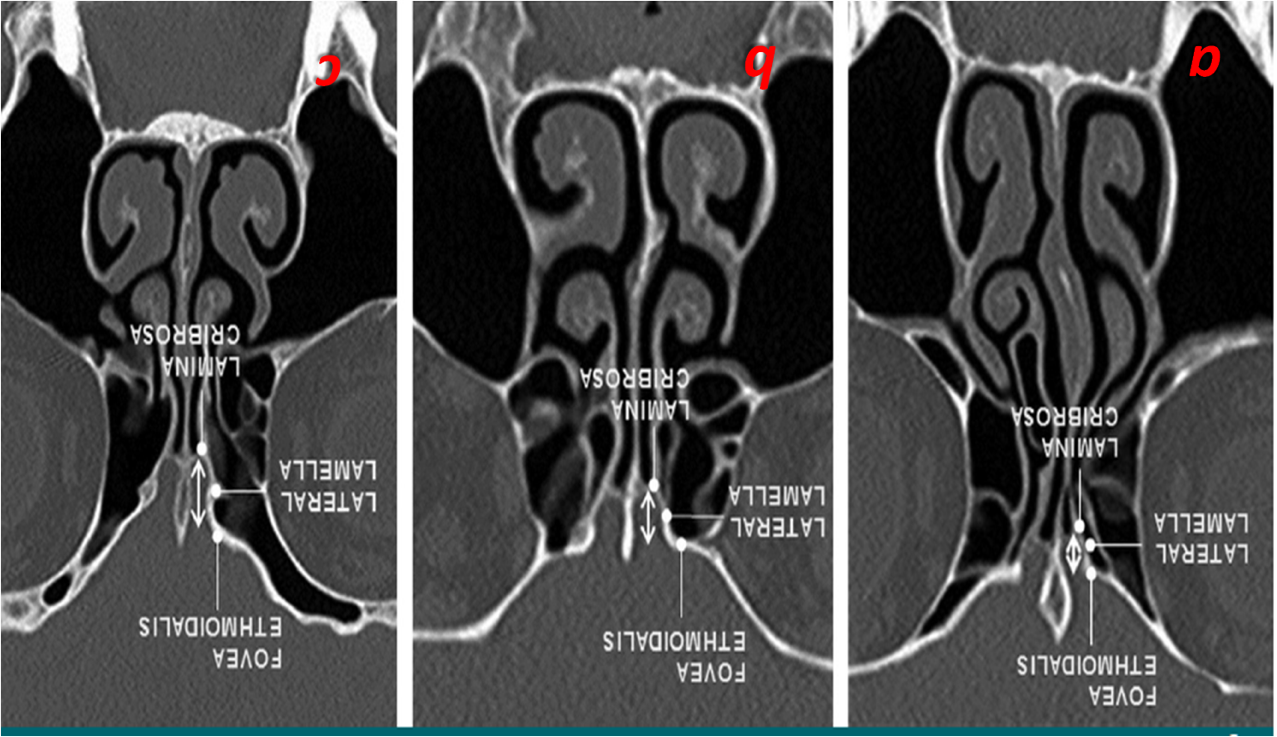 |
| Frontal cells (IFAC 2016 classification): | 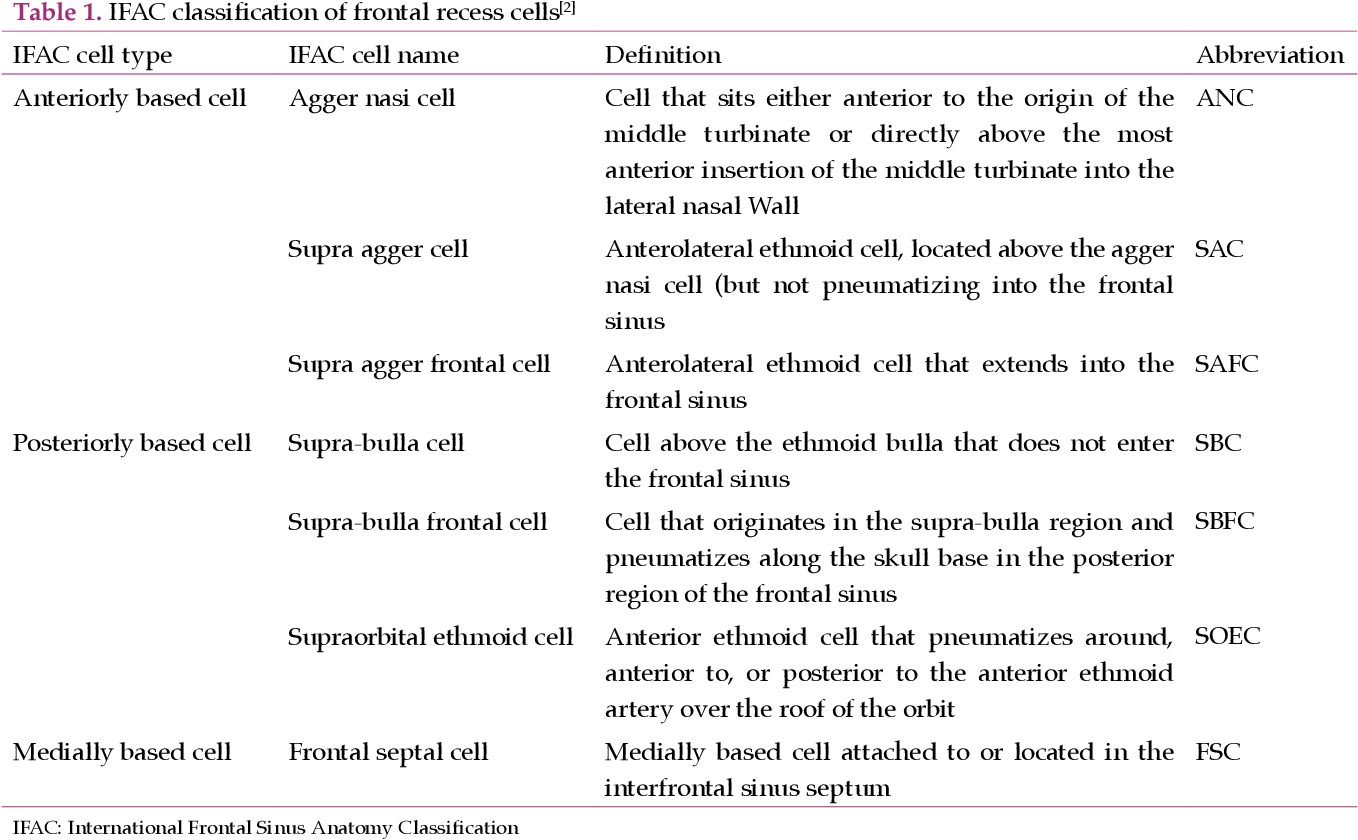 |
| Frontal recess:   - Inverted funnel shape that communicates with frontal sinus - Borders: medially: middle turbinate, laterally: lamina papyracea, posteriorly: ethmoid bulla, anteriorly: agger nasi | 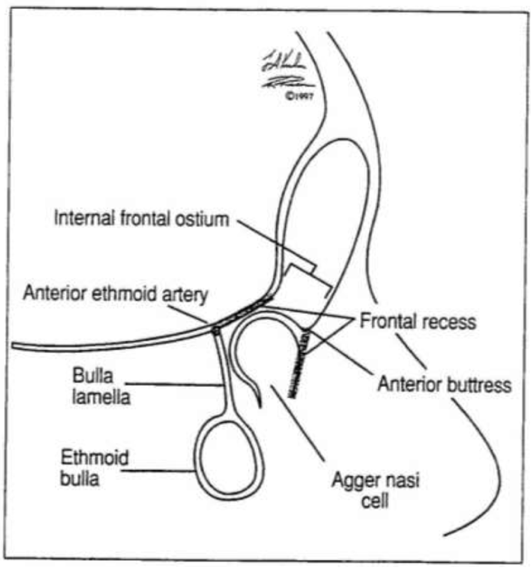 |
| Paranasal sinus mucociliary clearance | 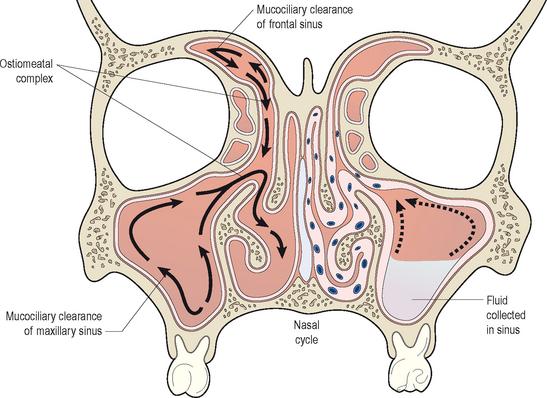**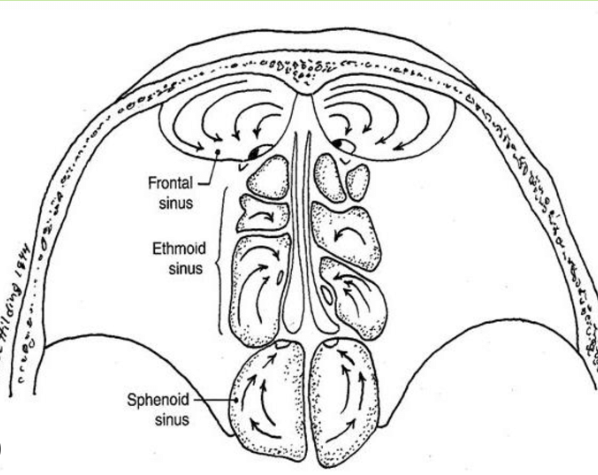** |
| Other anatomic structures of note:   - Agger Nasi cell: Superior and anterior to the uncinate process. The most anterior ethmoid cell remnant of the 1^st^ ethmoturbinal. - Haller cell (Infraorbital ethmoid cell): ethmoid air cell along the maxillary sinus roof / orbital floor - Onodi cell (Sphenoethmoidal cell) - Concha Bullosa: Pneumatized middle turbinate - Suprabullar recess: space between roof of ethmoid bulla and skull base - Retrobullar recess: space behind ethmoid bulla - Sphenoethmoidal recess: space between superior turbinate, face of sphenoid, and septum | 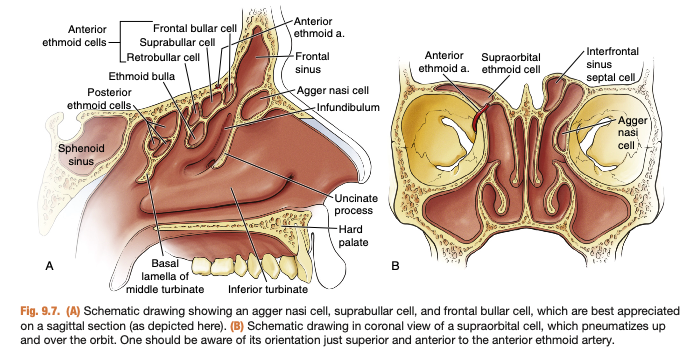 |

**3.0: FESS Instruments & room set-up**

- 3.5-mm straight non–through-cut forceps (Straight Blakesley) (A)
- 3.5-mm straight through-cut forceps (Straight Through Cut) (B)
- 3.5-mm upbiting non–through-cut forceps (45 degree Blakesley) (C)
- 3.5-mm upbiting through-cut forceps (D)
- Cottle periosteal elevator (45 degree Through Cut) (E)
- Ostium seeker or ball probe (F)
- 4-mm-long curved suction (G)
- Frazier suction (H)
- 360-degree sphenoid punch or forceps (I)
- 360-degree backbiting forceps (J)
- Kerrison
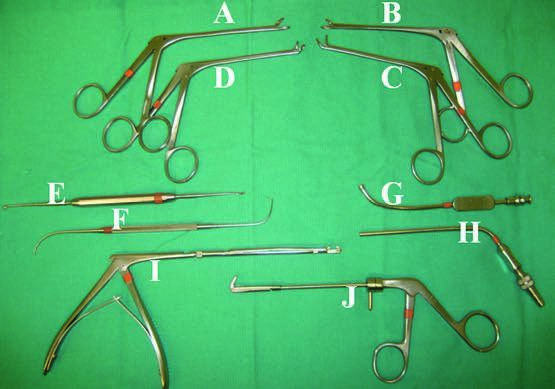


**4.0: Step-by-step FESS**

**Uncinectomy**

1. Begin with a zero-degree scope


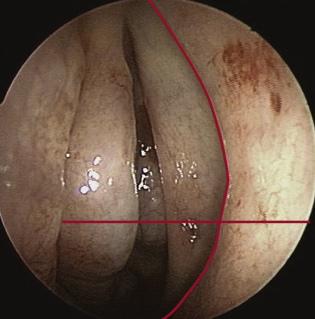


1. Using a Freer elevator, in-fracture and then out-fracture the inferior turbinate.
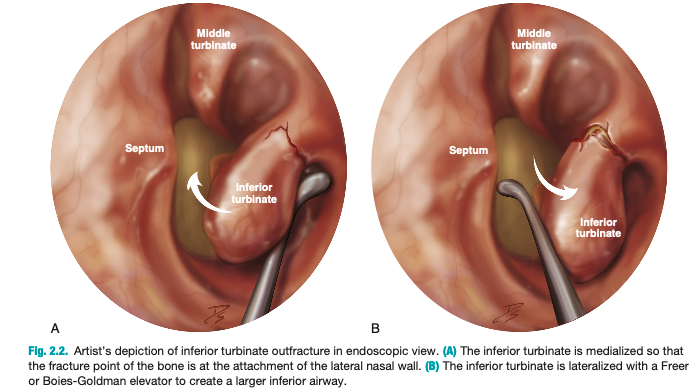

2. Using a Freer elevator, gently medialize the middle turbinate.


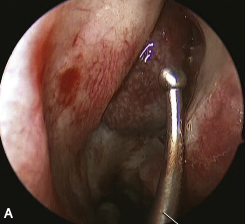


1. Identify free edge of uncinate process and using a ball-tip seeker, reflect the uncinate process anteriorly. This will lift it from the lamina papyracea and prevent injury


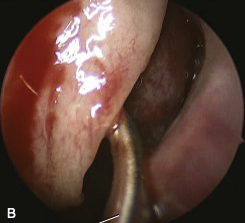


1. Introduce a backbiter in the closed position into the middle meatus, rotated so the biting blade opens upward in the vertical plane of the meatus. Open the blade and rotate horizontally to engage the posterior free edge of the uncinate process. Use the backbiter to remove the middle portion of the uncinate process from posterior to anterior, until you reach the maxillary line (green line).


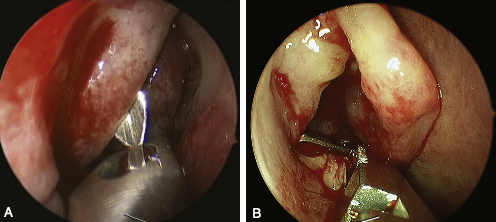


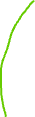

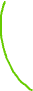


1. Use a 45 degree or 90 degree up-biting forceps to remove the superior attachment of the uncinate process all the way to the middle turbinate axilla
2. Remove the inferior uncinate process using microdebrider


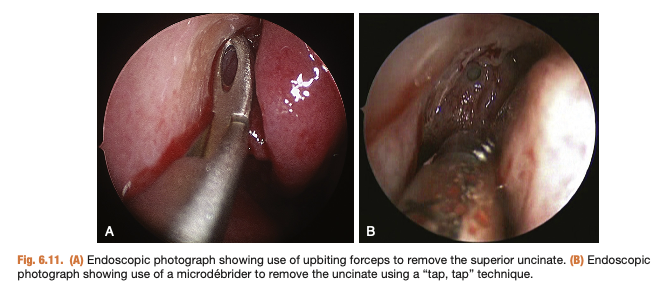


**Maxillary antrostomy (It is best to use an angled scope to maximize visualization)**

1. Use a ball-tip seeker to identify the natural os of the maxillary sinus – this should be easy to “fall into”. The angle of this is lateral and slightly inferior. Do not force a false passage as the risk of injury to the orbit is high.
2. Enlarge the antrostomy inferiorly by pushing the elbow of the ball-tip seeker inferiorly and posteriorly – this maneuver should result in an enlarged opening.
3. Use a down-biting antrum punch forceps to further enlarge the antrostomy inferiorly. Use a combination of the straight throughcut and/or microdebrider to clear the cut bone/mucosa. Do not strip the mucosal lining of the maxillary sinus.


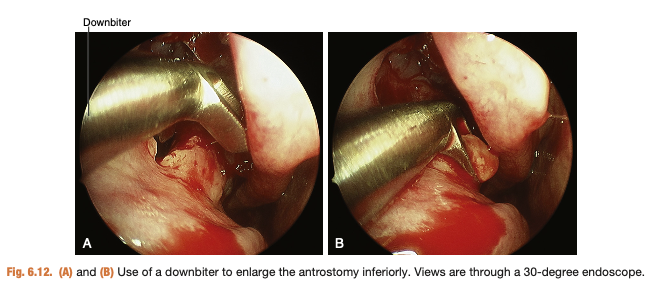

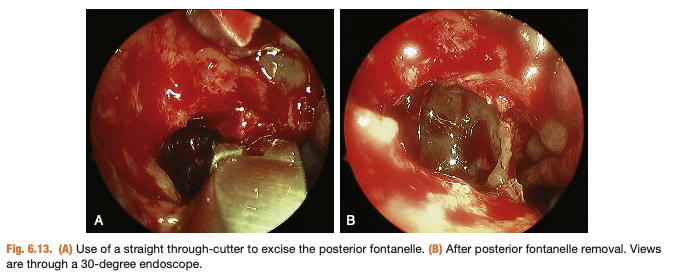


1. You can use a 30-degree scope to better visualize the maxillary antrostomy and debride any maxillary polypoid tissue


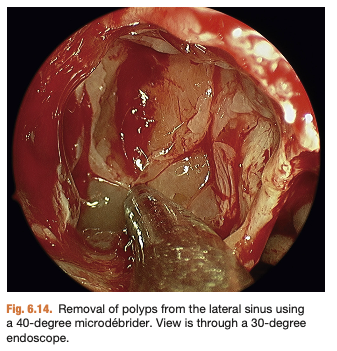


**Anterior and posterior ethmoidectomy**

1. Use a zero degree scope


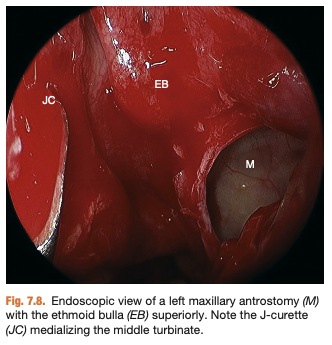


1. Identify the ethmoid bulla, and slide a J-curette behind the ethmoid bulla, into the retrobullar recess. Fracture the ethmoid bulla anteriorly.


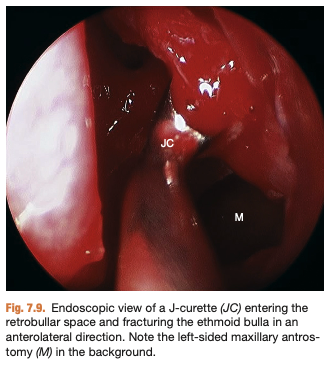


1. Use a microdebrider or up-biting through cut to debride the bony fragments. Do not strip mucosa


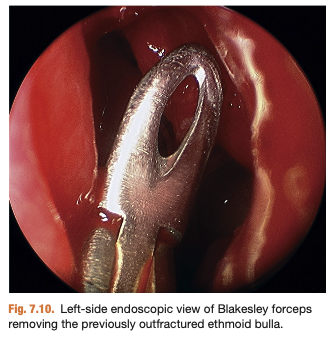


1. Continue to use the J-curette or up through-cutting forceps to palpate and fracture bony partitions in the anterior ethmoid, before debriding with the microdebrider. The goal is to clear all bony partitions between the middle turbinate/basal lamella and the lamina papyracea, without violating the lamina or skull base.
2. Identify roof of maxillary sinus – this level will guide you entry point through the basal lamella of the middle turbinate, and later on will approximate the level of the sphenoid os.


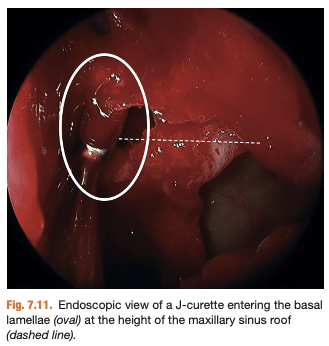


1. Perforate medial-inferior aspect of the basal lamellae of the middle turbinate at the level of the maxillary sinus roof using a J-curette. Use the microdebrider or upbiting through cut to take down the basal lamella of the middle turbinate, exposing the posterior ethmoid.
2. Identify the superior turbinate.


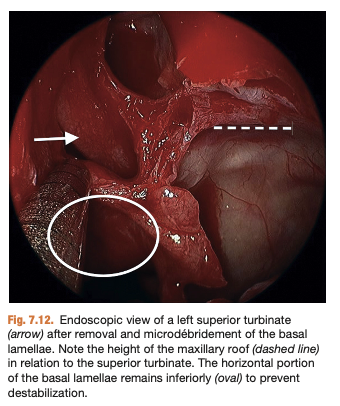


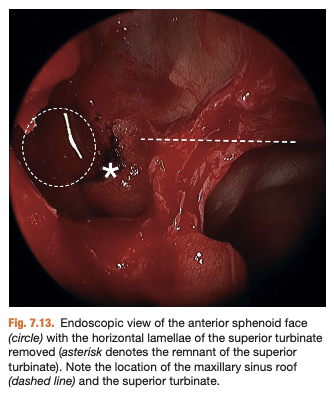


1. Use angled through cutting instruments to remove lateral partitions of the ethmoid cells off the lamina papyracea. Dissect the posterior ethmoid until the anterior face of sphenoid sinus is reached.
2. Identify the skull base superiorly in front of the sphenoid face and dissect posterior to anterior along the skull base using up-biting forceps (best) and minimize use of microdebrider. Be cognizant of the location of the anterior ethmoid artery during this step.


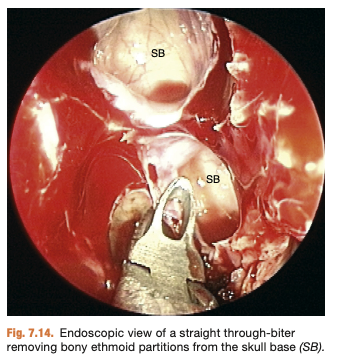

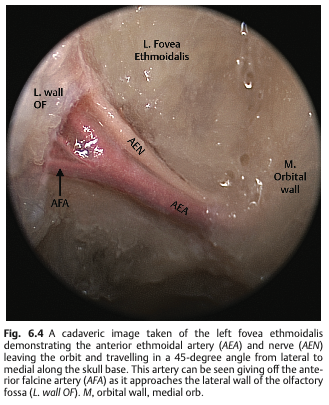

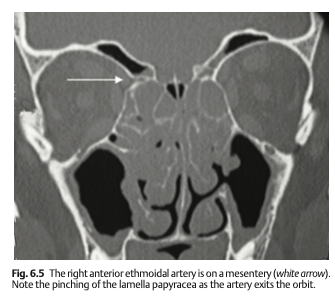


**Sphenoidotomy: anatomy**


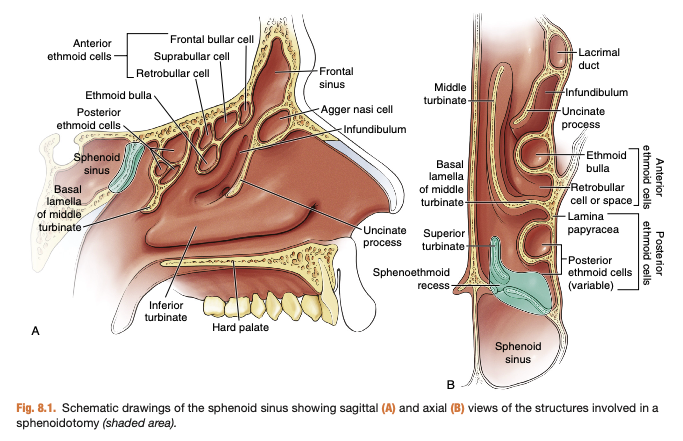
**
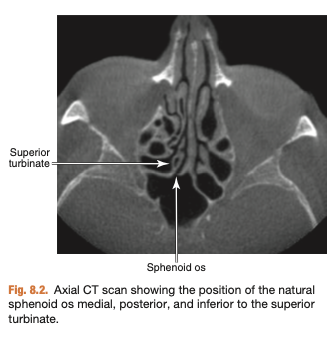
**
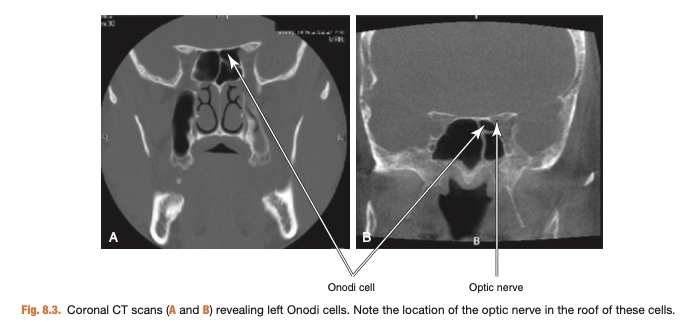


**Sphenoidotomy: anatomy**

1. Use a zero degree scope.
2. Identify the superior turbinate. Lateralize or remove lower third of superior turbinate using a straight through-cut or microdebrider.


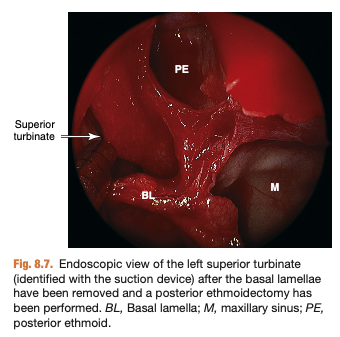

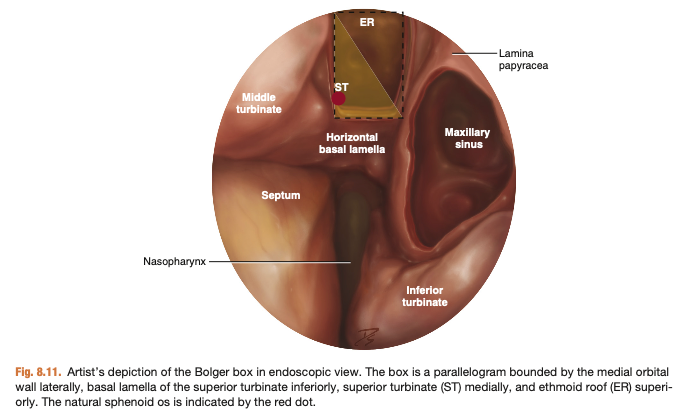


1. Identify natural os, which is usually behind/medial to the superior turbinate
2. Carefully use a J-curette or cottle elevator to enter and gently enlarge the os.
3. Use a straight mushroom punch, Kerrison or angled through cut to enlarge the os in an superior and lateral direction.
   1. Bolger’s Parallelogram/Box
      1. Lateral = medial orbital wall
      2. Medially = superior turbinate
      3. Inferiorly = basal lamella of the superior turbinate
      4. Superiorly = ethmoid roof

Tip: If you can see the floor of the cell when you are performing a sphenoidotomy, you are probably not in the sphenoid but in fact in a posterior ethmoid or Onodi cell


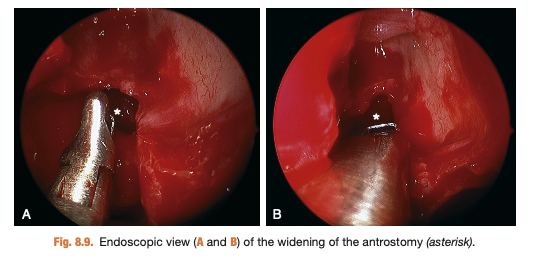

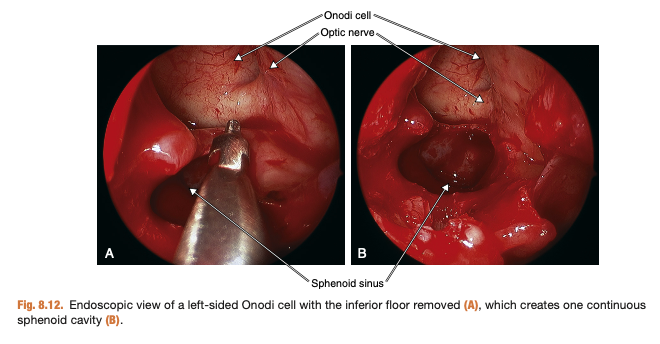


**5.0 Frontal Sinusotomy – Draf I and IIa/b**

**Draf I**

**
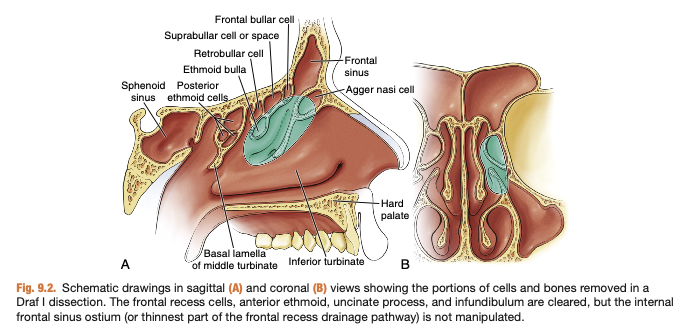
**

**Draf IIa**

**
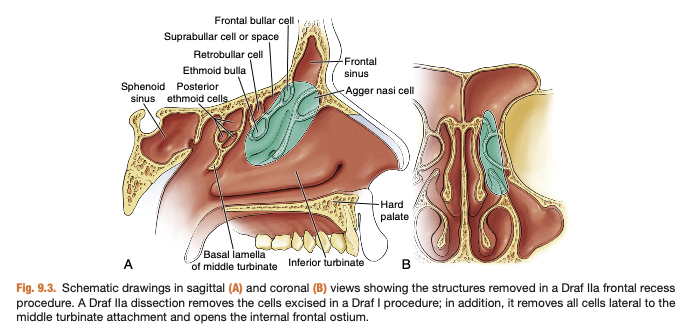
**

**Draf IIb**

**
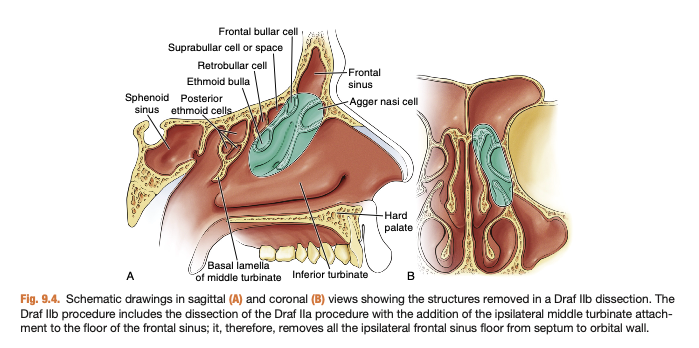
**

**Draf III**

**
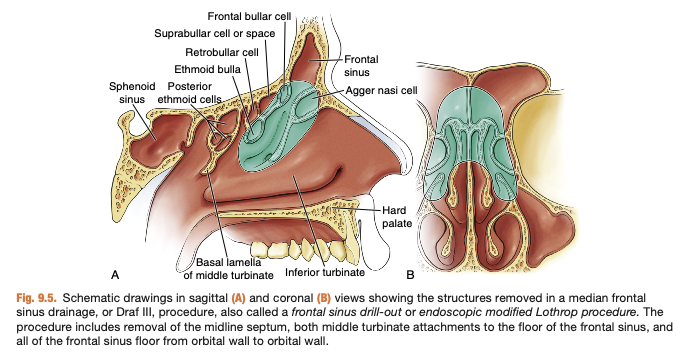
**

**Draf IIa frontal sinusotomy:**

1. Start with a 30-degree reverse endoscope and identify the anterior ethmoid artery. This is your posterior limit of your frontal sinus dissection.


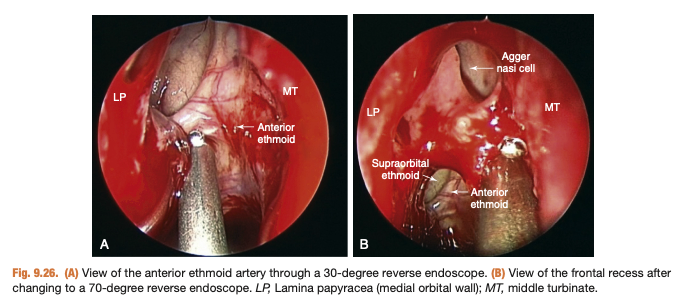


1. Identify the “transition zone” which is the natural drainage pathway of the frontal sinus that is medial-posterior to the posterior wall of the agger nasi cell. Identify the posterior wall of the agger nasi cell. Use a 45-degree front to back through-cutting giraffe forceps to remove the posterior wall of the agger nasi cell. You may consider using an angled curette to fracture the posterior wall of the agger nasi forward


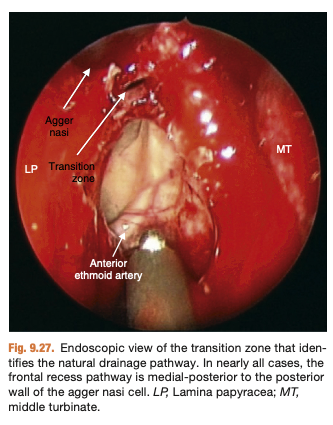

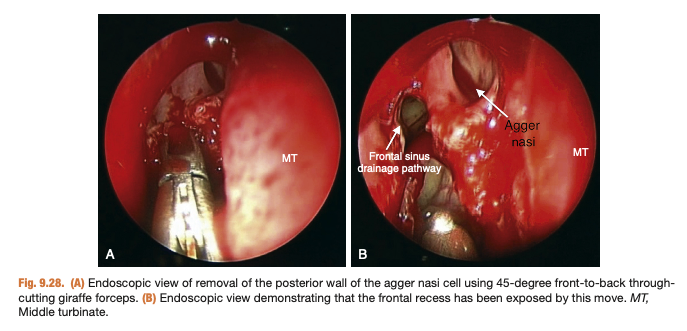


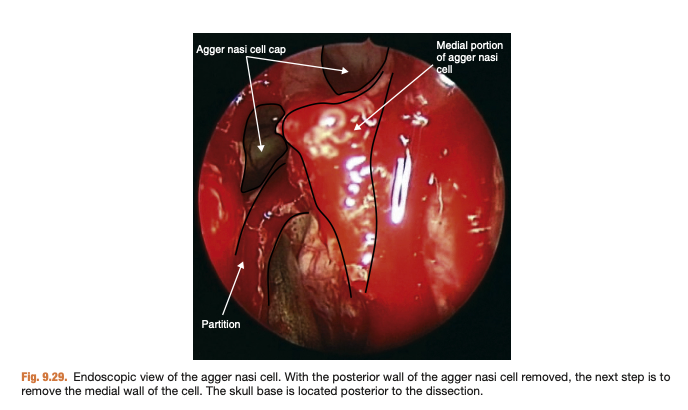

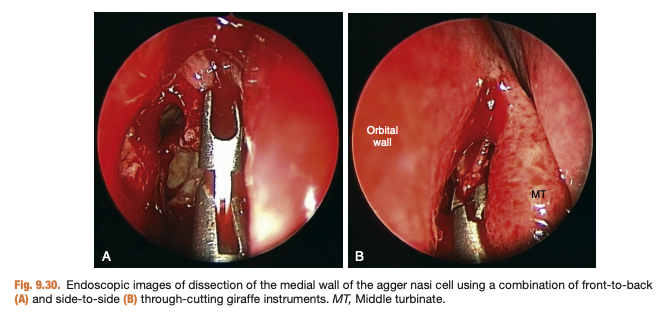


1. Remove surrounding bony partitions to widen the frontal recess. You can use a combination of front-to-back and side-to-side through-cutting giraffe forceps to cut the medial wall of the agger nasi cell and frontoethmoidal cells. Be careful not to violate the lamina papyracea or skull base, and avoid stripping mucosa.
2. Removing the cap of the agger nasi cell. Remove the cap of the agger nasi all the way anteriorly to the hard shelf of the nasofrontal bone.


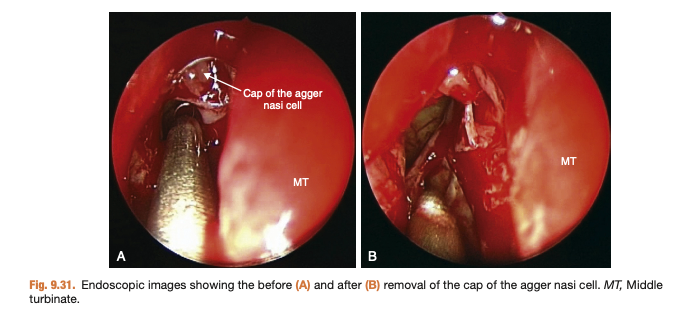


1. Expand the frontal recess posteriorly by removing the bony wall separating the supraorbital ethmoid and frontal recess using a 45-degree mushroom punch or a though-cutting giraffe forceps.
2. Expanded the frontal recess medially and laterally with a 45-degree mushroom or Hosemann punch.

***Draf IIb and Draf III procedures are not covered in this dissection manual
