# Supplementary material for: Validating the Calgary Simulation Curriculum: A Retrospective Review of Face and Content Validity of a Surgical Simulation Curriculum in Otolaryngology—Head and Neck Surgery
Source: J Otolaryngol Head Neck Surg. 2026 Apr 27;55:19160216261443996. doi: 10.1177/19160216261443996 (PMC13133485; doi:10.1177/19160216261443996)
Supplement: sj-docx-6-ohn-10.1177_19160216261443996 – Supplemental material for Validating the Calgary Simulation Curriculum: A Retrospective Review of Face and Content Validity of a Surgical Simulation Curriculum in Otolaryngology—Head and Neck Surgery [file sj-docx-6-ohn-10.1177_19160216261443996.docx]

**Rhinoplasty dissection manual**

**Objectives**

1. Describe the anatomy of the nose, including critical areas for nasal support (minor and major tip support structures).
2. Outline the surgical techniques and approaches to rhinoplasty.
3. Describe the indications of open and closed rhinoplasty approaches.
4. Outline the incisions required for open rhinoplasty and indications for each.
5. Understand the role and indication for osteotomies in rhinoplasty surgery.

**Brief anatomy**

| **Internal nasal valve** | - Septum - Caudal ULC - Inferior turbinate   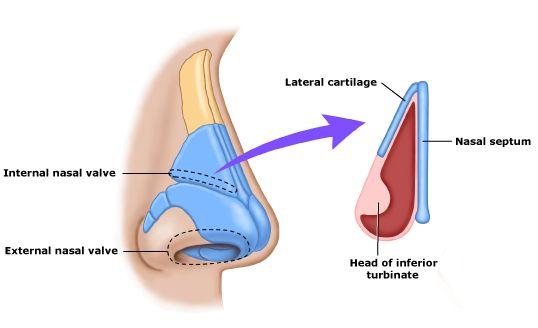 |
| --- | --- |
| **Major tip support structures** | - Size shape & resiliency of LLC - Connection of LLC medial footplate to caudal septum - Connection of LLC & ULC (scroll area) |
| **Minor tip support structures** | - Dorsal septum - Inter-domal ligaments - Membranous septum - Nasal spine - Skin & soft tissue envelope - Alar sidewalls - Sesamoid cartilage |

| **Surgical approaches**  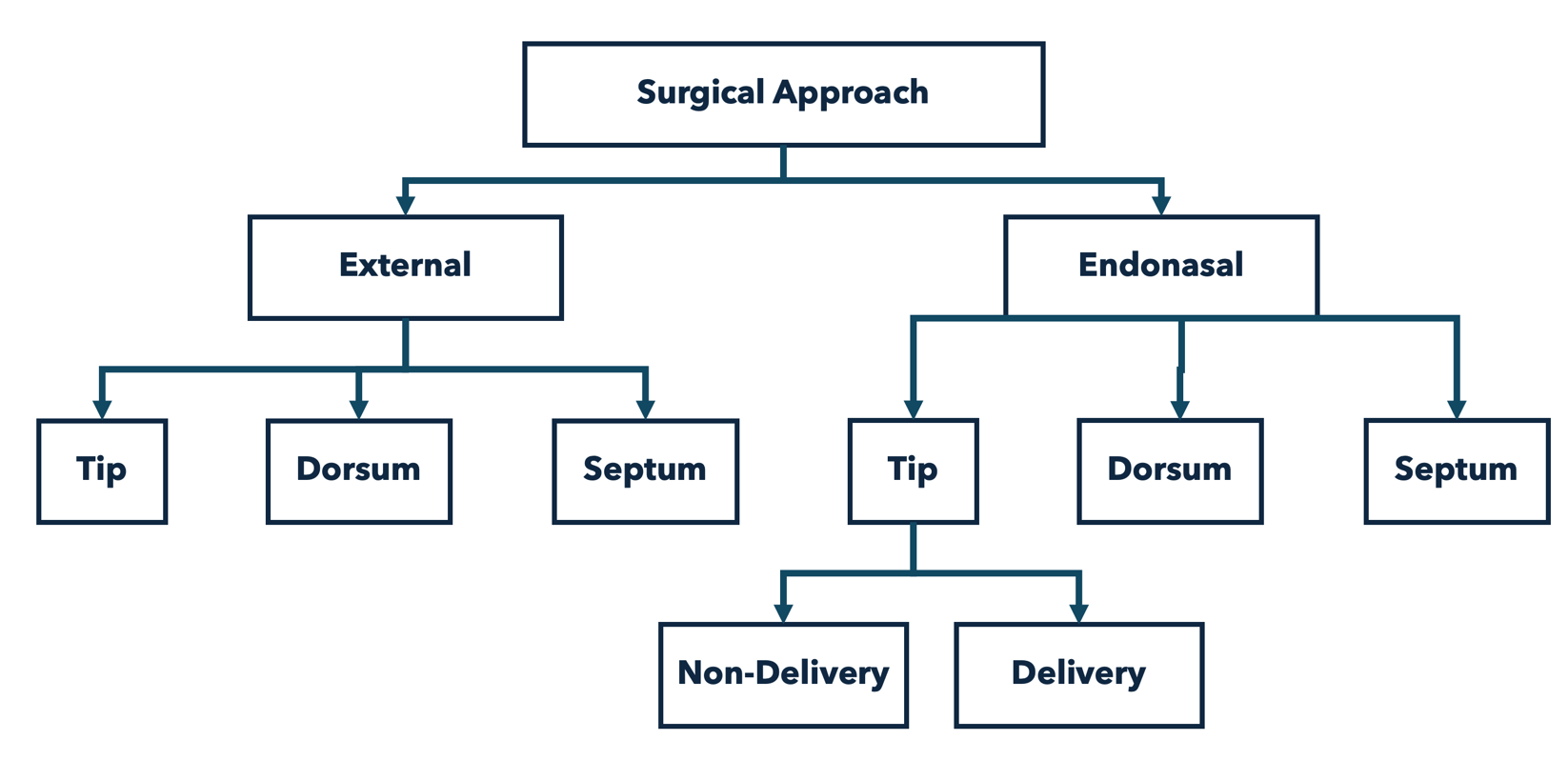 | | |
| --- | --- | --- |
| **Endonasal** | | |
| **Non-delivery Approach** | | |
|  | **Advantages:**   - No major tip support affected - Preserves intact caudal rim - One incision - No external scar - Reduces surgical trauma and edema | **Disadvantages:**   - Minimal exposure of LLC - Limited to conservative tip work - Risk of asymmetrical incisions |
| **Cartilage splitting (trans/intercartilagenous)** | Indicated for minimal refinement of nasal tip | |
| **Retrograde approach** | For minimal refinement of nasal tip with thick skin, uses an intercartilaginous incision | |
| **Delivery Approach**   - Combines intercartilaginous and marginal incisions - Allows delivery of LLC as a bilateral pedicles chondrocutaneous flap | | |
| **Advantages:**   - Visualizes entire LLC - Good access to tip and nasal dome - No external scar | | **Disadvantages:**   - Compromises major support by removing the attachment of the alar cartilages and the ULC - Causes more edema (post-op distortion) - Requires 2 incisions |

| **Indications for Open Rhinoplasty Approach** | | |
| --- | --- | --- |
| **Medical** | **Cosmetic** | **Other** |
| - Closure of septal perf - Cleft lip or palate rhinoplasty - Saddle nose deformity - Nasal tumour | - Crooked nose lower 2/3 - Eccentric/asymmetric - Extensive tip work - Major reconstruction - Short nose requiring caudal extension | - Teaching - Surgeon preference - Non-Caucasian nose |

| **Order of open approach:**   1. Exposure 2. Septum 3. Dorsum modification/reduction 4. Osteotomies 5. Spreader grafts 6. Tip work +/- grafts 7. Closure 8. Alar base modification (weir excision) | |
| --- | --- |
| **Advantages:**   - Better exposure - Undistorted view of anatomy - Better diagnosis of distorted anatomy - Easier to execute maneuvers, place sutures and grafts, trim cartilage exactly, excise scar tissue - Easier to protect internal nasal valve | **Disadvantages:**   - Columellar scarring - Poor exposure - Longer lasting swelling |

| **Open Rhinoplasty Incisions** | | |
| --- | --- | --- |
| T**rans-columellar incision** | - Transverse incision of the **skin** of the columella - Above the feet of the medial crura - Below the feet of the medial crura, there is no cartilaginous skeleton to resist scar tension. - Used in open rhinoplasty | 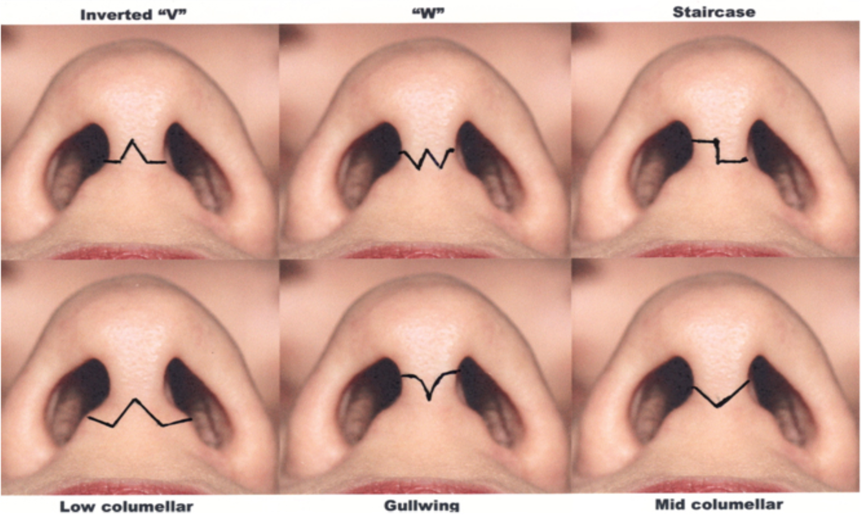 |
| **Marginal/rim** | - At the caudal border of the LLC - Made parallel to the caudal borders of the LLC - End where the caudal edges of the lateral crura diverge cephalically away from the rim - More stability for grafts | 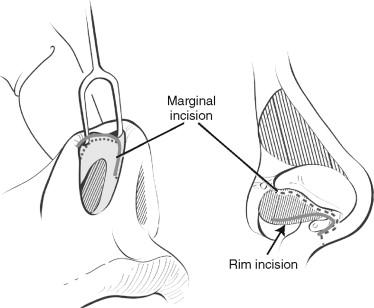 |
| **Intercartilagenous** | - Access to tip and mid nose - Intranasal incision between the ULC and LLC - Begin medially as transfixion extension - Continue entire length of LLC - Avoid transecting the lateral end of the LLC | 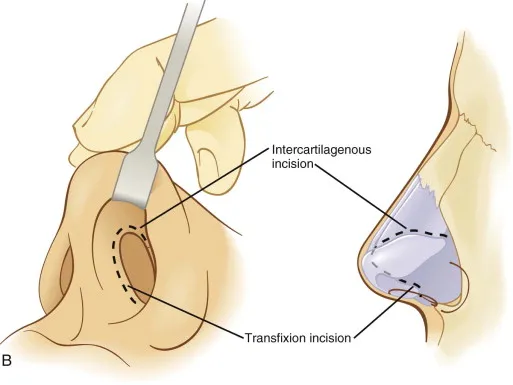 |
| **Infracartilaginous** | - Access to the tip and mind nose - Incise through the vestibular mucosa +/- LLC - Similar to intercartilaginous but 3-5mm caudal to the cephalic end of LLC to prevent knuckles or bending of the weak cartilage - Caudal to nasal valve | 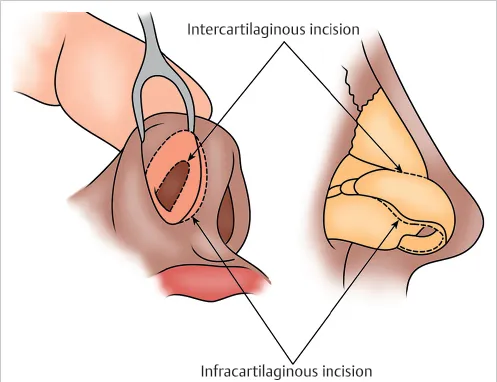 |

| **Approaches to Specific Deformities** | | | | |
| --- | --- | --- | --- | --- |
|  | **Graft technique** | **Cartilage technique** | **Suture technique** | **Other** |
| **Increase tip projection**  *Causes:*   - *Hypoplastic nasal spine/maxilla* - *Short septum* - *Short/weak medial crura* - *Posterior attachment of medial crural to septum* | - Shield graft - Columellar strut graft - Plumping graft columellar labial angle | - Lateral crural steal - Vertical dome division (Goldman) - Tongue in groove (move anterior on septum) | - Transdomal sutures - Intradomal sutures - Tongue-in-groove (suture medial crura more anteriorly onto the caudal septum) | - Plumping graft (placed at the base of the columella and changes the columellar-labial angle with an illusion of increased projection) |
| **Decrease tip projection**  *Causes:*   - *Prominent nasal spine* - *Prominent septum* - *Long medial crura* - *Anterior attachment of medial crura to septum* |  | - Complete transfixion of caudal septum - Dome division - Shorten lateral or medial crura - Nasal spine reduction - Detach ULC from LLC - Interrupted strip of medial crura - Septal excision | - Tongue-in-groove (suture medial crura more posteriorly onto the caudal septum) |  |
| **Increase tip rotation**  *Causes:*   - *Laxity of ULC/LLC* - *Long ULC* - *Caudal position of LLC on septum (too much tip for septum)* - *Long lateral crura relative to medial crura* | - Columellar: strut graft | - Cephalic trim - Shorten ULC - Lateral crural division + overlay - Septal: inverted wedge of cartilage septum (top) | - Tongue in groove medial crura on septum | - Reduce nasal dorsum - Plumping graft |
| **Decrease tip rotation**  *Causes:*   - *Short ULC* - *Long medial crura relative to lateral crura* - *LLC positioned high on septum* | - Detach ULC & LLC - Complete/interrupted strip medial crura - Dome division + reposition - Infratip onlay shield graft - Dorsum onlay graft | - Trim a wedge of septum inferiorly (near the nasal spine) | - Tongue in groove: Suture the medial crura posteriorly |  |
| **Tip definition**  *Causes:*   - *Thick skin* - *Large lateral crura* - *Wide interdomal distance* | - Cephalic trim for bulbous - Vertical dome division - Onlay tip graft - Suture: intradomal, interdomal (below), double dome sutures (both) |  |  |  |
| **Columellar show / alar retraction** | **Management of alar retraction:**   - Alar rim graft - Alar batten graft - Lateral crural repositioning | | **Management of hanging columella:**   - Excision of caudal septum - Excision of membranous septum - Excision of caudal medial crura | |

| **Osteotomies**  Always move medial to lateral   - Straighten a crooked nose - Flatten convex nasal bones - Reduce open roof defect caused by removal of a dorsal hump   - Most are from cartilaginous septum   - Nasal skin is thinnest at the mid dorsum (rhinion) therefore must create a slight mid-dorsal hump to avoid saddle nose deformity | | |
| --- | --- | --- |
| **Medial** | **Intermediate** | **Lateral** |
| - Used to free nasal bones from perpendicular plate of ethmoid | - Used to correct deviated nasal dorsum or straighten excessively wide or convex nasal bones | - Start anterior and superior to the head of the inferior turbinate - Can also be done transcutaneously using a 2mm straight osteotome |
