# Supplementary material for: Validating the Calgary Simulation Curriculum: A Retrospective Review of Face and Content Validity of a Surgical Simulation Curriculum in Otolaryngology—Head and Neck Surgery
Source: J Otolaryngol Head Neck Surg. 2026 Apr 27;55:19160216261443996. doi: 10.1177/19160216261443996 (PMC13133485; doi:10.1177/19160216261443996)
Supplement: sj-docx-7-ohn-10.1177_19160216261443996 – Supplemental material for Validating the Calgary Simulation Curriculum: A Retrospective Review of Face and Content Validity of a Surgical Simulation Curriculum in Otolaryngology—Head and Neck Surgery [file sj-docx-7-ohn-10.1177_19160216261443996.docx]

┌───────────────────────────────────────────────────────────┐
│ **CALGARY SIMULATION CURRICULUM (CSC)**  │
│ Annual, Multi-Session Simulation Program (5 Sessions) │
└───────────────────────────────────────────────────────────┘
┌───────────────────────────────────────────────────────────┐
**│ CURRICULUM DEVELOPMENT** │
│ • Identified training gaps │
│ (resident–faculty program committee discussions) │
│ • Resident consensus on utility │
│ • Alignment with RCPSC EPAs │
└───────────────────────────────────────────────────────────┘
┌───────────────────────────────────────────────────────────┐
**│ PREPARATION PHASE** │
│ • Session-specific learning objectives │
│ • Preparatory materials (readings, manuals, images) │
│ • Distributed in advance │
└───────────────────────────────────────────────────────────┘
┌───────────────────────────────────────────────────────────┐
│ **SIMULATION DELIVERY (3 HOURS / SESSION)** │
│ Learner Structure: │
│ • Junior residents (PGY 1–2) │
│ • Senior residents (PGY 3–5) │
│ • Faculty facilitators │
│ Teaching Model: │
│ • Faculty-led demonstration │
│ • Senior resident near-peer instruction │
│ • Junior resident hands-on performance │
└───────────────────────────────────────────────────────────┘
┌───────────────────────────────────────────────────────────┐
│ **FIVE CORE SIMULATION SESSIONS** │
│ │
│ 1. Pediatric Airway Foreign Body (Mannequin-based) │
│ 2. Neck Dissection (Cadaveric, Levels I–IV) │
│ 3. Functional Endoscopic Sinus Surgery (Cadaveric) │
│ 4. Nasal Fracture & Sphenopalatine Artery Ligation │
│ (Cadaveric) │
│ 5. Rhinoplasty (Cadaveric) │
└───────────────────────────────────────────────────────────┘
┌───────────────────────────────────────────────────────────┐
**│ EVALUATION & FEEDBACK** │
│ • Post-session resident surveys │
│ • Educational value │
│ • Teaching quality (faculty & residents) │
│ • Utility of preparatory materials │
│ Outcomes Used to: │
│ • Assess face validity │
│ • Guide curriculum refinement │
└───────────────────────────────────────────────────────────┘

**Figure Caption:** Visual schematic illustrating the development, structure, delivery, and evaluation of the Calgary Simulation Curriculum (CSC), a multi-session, competency-aligned simulation program for Otolaryngology–Head and Neck Surgery residency training. The curriculum is delivered annually during protected academic time, incorporates a mixed faculty–resident teaching model, and undergoes iterative refinement based on learner feedback.
